# UKGrsHP: a UK high-resolution gauge–radar–satellite merged hourly precipitation analysis dataset

**DOI:** 10.1007/s00382-020-05144-2

**Published:** 2020-02-07

**Authors:** Jingjing Yu, Xiao-Feng Li, Elizabeth Lewis, Stephen Blenkinsop, Hayley J. Fowler

**Affiliations:** School of Engineering, Newcastle University, Newcastle upon Tyne, NE1 7RU UK

**Keywords:** Hourly precipitation, Optimal interpolation, Multi-source data merge, UK, High resolution

## Abstract

There is an urgent need for high-quality and high-spatial-resolution hourly precipitation products around the globe, including the UK. Although hourly precipitation products exist for the UK, these either contain large errors, or are insufficient in spatial resolution. An efficient way to solve this is to develop a merged precipitation product that combines the information and benefits from multiple data sources, improving both the spatial resolution and accuracy of hourly precipitation estimates over the UK. In this study, we develop a UK high-resolution gauge–radar–satellite merged hourly precipitation analysis: the UKGrsHP. It covers the UK from 12.5° W to  3.5° E, 49° N–60° N, with a spatial resolution of 0.01° × 0.01° in latitude/longitude (equivalent to 1 km resolution in the mid-latitudes). An optimal interpolation (OI)–based multi-source merging scheme with compound strategy is developed and tested for producing the UKGrsHP. Three input data sources are used: gauge analysis data interpolated from 1903 quality-controlled hourly observations, the UK Nimrod radar precipitation analysis and the GSMaP global satellite precipitation analysis. Using independent tests against ~ 220 independent gauge observations on 1 year’s experimental UKGrsHP, covering the period from January to December 2014, we find that the final merged data performs better than three individual precipitation analyses used as inputs. A full version of the UKGrsHP starting in April 2004 is now under production, which will have wide applications in climate services and scientific research across multiple disciplines.

**Electronic supplementary material:**

The online version of this article (10.1007/s00382-020-05144-2) contains supplementary material, which is available to authorized users.

## Introduction

The urgent need for high-resolution precipitation products (HRPs) arises from both public services and scientific research, with increased occurrence of extreme rainfall events (e.g., Kendon et al. 2014; Archer and Fowler 2015) and flash floods (e.g., Westra et al. 2014) under a warming climate. The development of HRPs have been mainly based on three types of observational precipitation data sources: (1) rain gauge observations, which can provide accurate point rainfall estimates, but whose spatial resolution is limited by the low-density gauge network and the errors associated with interpolation schemes to infill missing data (e.g. Morrissey et al. 1995; Xie et al. 2007; Chen et al. 2008); (2) satellite estimation, which has wide spatial coverage but relatively poor precision (e.g. Hong et al. 2007; Tian et al. 2009); (3) radar quantitative precipitation estimation (QPE), which has very high spatial and temporal resolution, but whose accuracy is lower than ground rain gauge observations and whose spatial coverage is restricted by the availability of the operational radar network (e.g., Maddox et al. 2002; Vasiloff et al. 2007). An efficient way to develop HRPs is to merge precipitation data sources, thus extracting the useful information from different data types (including the above and even analysis/reanalysis data), then merging them together into a new precipitation data product. Successfully merged precipitation data is generally of better quality than its individual input data sources. Due to these advantages, the development of merged precipitation products has attracted more and more attention in recent years.

Early efforts developed various global merged precipitation products at relatively coarse spatiotemporal resolutions, mainly through the merging of gauge-based analyses and satellite estimates (Adler et al. 2003). Widely recognized and applied global merged precipitation analysis datasets include the Climate Prediction Centre (CPC) Merged Analysis of Precipitation (CMAP) (Xie and Arkin 1997) and the Global Precipitation Climatology Project (GPCP) precipitation analysis (Adler et al. 2003). The CMAP and the GPCP were constructed at both monthly and pentad time resolutions on a global domain at a spatial resolution of 2.5° × 2.5°, spanning 1979 to present. They merge multiple satellite estimates and the GPCC gauge analysis, but using different methods. Assessment studies (e.g., Ebert 1996; Adler et al. 2001; Yin et al. 2004) have shown that both the CMAP and the GPCP substantially reduce biases and random errors compared with those of individual gauge and satellite datasets. The GPCP has also developed a daily product (at 1° × 1°) from late-1996 to present, in which the satellite infrared (IR) is calibrated to the monthly GPCP amount and passive microwave (PMW) frequency of precipitation over 40° S–40° N, and sounding-based data at higher latitudes (Huffman et al. 2001). However, the temporal and spatial resolutions of the CMAP and the GPCP are relatively coarse for monitoring the diurnal and mesoscale features of heavy precipitation (Dai et al. 2007). Further efforts have therefore been made to increase the spatiotemporal resolutions of globally merged precipitation analyses. One recent example is the Multi-Source Weighted-Ensemble Precipitation (MSWEP), which takes advantage of the complementary strengths of gauge-, satellite-, and reanalysis-based data by optimally merging the highest quality precipitation data sources available as a function of timescale and location (Beck et al. 2017). The latest version (V2.2, released March 2, 2018) of MSWEP (1979–2017) has been improved to a 3-hourly temporal and 0.1° spatial resolution (Beck et al. 2019). So recent advances have improved the spatiotemporal resolutions of globally merged precipitation analyses from monthly to 3-hourly and from ~ 250 to ~ 10 km in the mid-latitudes, within an acceptable bias.

These globally merged precipitation analyses are suitable for large-scale analysis, but still have big gaps in satisfying the crucial requirements for regional applications, such as monitoring the diurnal and mesoscale features of heavy precipitation, driving high-resolution numerical weather (NWP) models or distributed hydrological models over regional domains, etc. Thus, extensive research has focused on generating regional and quantitatively accurate merged precipitation analysis datasets with fine spatiotemporal resolutions of 1 h to 5 min and 1–10 km. To attain a better spatiotemporal resolution than most global precipitation products, integrating information from radar precipitation products is widely used in producing regional merged precipitation analyses, as radar has the unique advantages of high spatial and temporal resolution. Satellite data is also valuable in regional precipitation merging, as it provides the precipitation signal over a much larger spatial domain than either gauge or radar data.

There are some well-known pioneering regional gauge-radar-merged hourly precipitation analyses. One is the Stage IV product (Lin and Mitchell 2005), which combines gauge and radar data. It is at hourly and 4 km resolution and covers the Continental United States (CONUS), produced at NOAA/NCEP through a mosaic procedure of Stage III bias-corrected radar precipitation products generated at individual NOAA River Forecast Centers. QPE products at very high spatial (1 km) and temporal (2 min) resolution that cover CONUS and southern Canada have been produced by the Multi-Radar Multi-Sensor (MRMS) system at the National Center for Environmental Prediction (NCEP) (Zhang et al. 2016). The MRMS QPE system integrates radar, rain gauge, satellite and atmospheric environmental and climatological data, and its QPE algorithms are largely based on the National Mosaic and Multi-Sensor QPE (NMQ) (Zhang et al. 2011) components from the National Severe Storms Laboratory (NSSL).

Near-real-time precipitation analyses have also been developed. For example, the Integrated Nowcasting through Comprehensive Analysis (INCA) precipitation analysis at an updated resolution of 15 min and 1 km has been developed in Europe. INCA incorporates station data, radar data, and elevation effects (Haiden and Pistotnik 2009), and was developed at the Central Institute for Meteorology and Geodynamics in Vienna, Austria (ZAMG). The INCA precipitation analysis is a successful attempt to combine the quantitative accuracy (compared to radar) of rain gauge measurements with the spatial accuracy provided by the radar field (Haiden et al. 2011).

Recently, the National Meteorological Information Centre of China (NMICC) developed a series of gauge-satellite and gauge–radar–satellite merged hourly precipitation products, at fine spatial–temporal resolutions of 10 km, 5 km and 1 km, covering the whole of China including the eastern Tibetan Plateau (Shen et al. 2014, 2018; Pan et al. 2015). The merging is based on the multi-source merging algorithm (Pan et al. 2012; Yu et al. 2013) developed from the two-step merging conceptual model of Xie and Xiong (2011). The NMICC gauge–radar–satellite merged Chinese hourly precipitation analyses exhibit greater accuracy than other hourly precipitation products for precipitation distributions and variability over China, largely attributed to the merging of high-density hourly precipitation gauge observations (more than 30,000 automatic weather stations: Shen et al. 2014; Yu et al. 2015). Satellite data is applied in the NMICC merged Chinese hourly precipitation analyses due to the lack of either radar or gauge data over much of western China and the Tibetan Plateau, where satellite estimation is the only precipitation data source, and also further improves the quality of final merged data, especially over sub-regions with sparse stations where the radar data has less chance to be corrected (Pan et al. 2018).

Over the UK, very few regional hourly precipitation products are available. Recently, two UK high-resolution hourly precipitation products have been released, mainly developed from a single precipitation source: (1) The NIMROD radar precipitation analysis data developed by the UK Met Office is available at 5 and 15 min intervals on a 1 km and 5 km Cartesian grid over UK, starting from late 2002. These fine-resolution radar precipitation analyses are produced by a fully automated system called Nimrod for weather analysis and nowcasting based around a network of C-band rainfall radars (Golding 1998) operated by the Met Office since 1996; (2) A 1 km gridded hourly gauge-based precipitation analysis (CEH-GEAR1hr) for the UK developed by Lewis et al. (2018) covers the period 1990–2014. It was derived from over 1900 quality-controlled rain gauges using a nearest neighbour interpolation scheme and improves upon the current UK national gridded precipitation datasets at the daily time-step (Blenkinsop et al. 2017; Lewis et al. 2018).

This paper aims at developing a UK high-quality hourly gauge–radar–satellite merged precipitation analysis—the UKGrsHP— by employing mature integrated algorithms and approaches (Xie and Xiong 2011; Shen et al. 2014) to make full use of existing multi-source UK precipitation measurements including gauge, radar and satellite data. An optimal integration (OI)-based multi-source merging scheme is designed and tested on independent gauge data. One year’s experimental UKGrsHP is then produced by this merging scheme, which not only has a fine spatial resolution of 0.01° × 0.01° in latitude/longitude but also has better performance than other existing hourly precipitation products over the UK. The evaluated OI-based multi-source merging scheme will be applied to produce the full version of the UKGrsHP starting in 2004.

The rest of the paper is organized as follows. Section 2 describes the data, basic OI theory, and methods of data evaluation and independent test used in this research. Section 4 introduces the flowchart of the data merging of the UKGrsHP. Sections  5, 6, 6.1 expound the implementation of the merging algorithm and its validation in detail of the three major steps of the data merging, including the Data Pre-processing (Step 1), the PDF systematic-bias-correction of radar analysis and satellite analysis data (Step 2) and the OI-based multi-source merging for the UKGrsHP (Step 3). Section 7 presents the results of independent evaluation of the experimental UKGrsHP. Finally, a summary and recommendations for future research are given at the end of the paper in Sect. 7.3.

## Data, OI theory, evaluation and independent test methods

### Information of three hourly precipitation datasets used in the study

Three hourly precipitation datasets are used in the UK data merging experiment: UK rain gauge observations, UK rain radar data, and a satellite-retrieved precipitation product. All three input data sources and the output merged data (the experimental UKGrsHP) cover the same one-year period from 1 January 2014 to 31 December 2014.

**Fig. 1:**
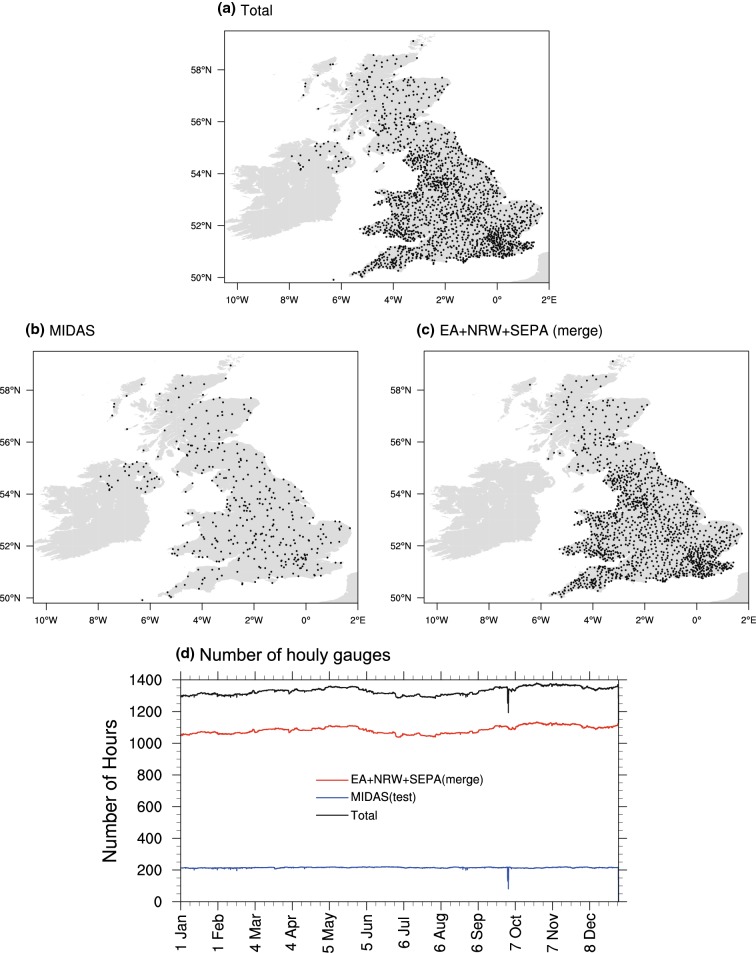
**a** The spatial distribution of all 1903 gauge stations over the UK (black points), **b** the 407 checking stations used for independent testing from MIDAS (black points), **c** 1496 stations taking part in the merging process from the EA, NRW and SEPA (black points), **d** the actual numbers of hourly gauge observations processed from hour to hour for total stations (black line), check stations (blue line) and the merging stations (red line) for the independent test over the UK from 1 January 2014 to 31 December 2014

We use 1903 quality controlled UK rain gauges (see Fig. 1a for coverage) from Blenkinsop et al. (2017) and Lewis et al. (2018). The original raw UK gauge data are from the UK Met Office Integrated Data Archive System (MIDAS, UK Met Office 2012), the England Environment Agency (EA), Natural Resources Wales (NRW) and the Scottish Environmental Protection Agency (SEPA). As the measurements of precipitation gauge at instrument sites contain inevitable observational errors (McMillan et al. 2012), various quality control procedures were applied by Blenkinsop et al. (2017) and Lewis et al. (2018) to identify suspect values, accumulated totals and periods of gauge malfunction to get high-quality rain gauge data. These quality-controlled rain gauges are also available in the global sub-daily rainfall (GSDR) dataset (Lewis et al. 2019) collected by the INTENSE project (Blenkinsop et al. 2018). It should be noted that the actual number of gauges participating in the merging process at any one time-step is less than 1903 and this changes from hour to hour throughout the year, with an average of around ~ 1300 gauge records per hour, as shown by the black line in Fig. 1d. This is due to missing data, some short data records and the quality control procedure detailed above.

The UK Nimrod radar data are from the Met Office’s operational Nimrod system (Harrison et al. 2000). Here we use the 1 km Resolution UK Composite Rainfall Data downloaded from https://catalogue.ceda.ac.uk/uuid/27dd6ffba67f667a18c62de5c3456350 (Golding 1998; Harrison et al. 2000). This dataset is originally sourced from a network of 15 C-band rainfall radar sites, starting in April 2004 and updated in real-time. It is produced at 5 min intervals on a 1 km resolution grid on the British National Grid (BNG) reference system (Ordnance Survey 2008), covering the rectangle from – 405,000° E to – 625,000  N and 132,000° E to 155,000  N (an area of 3.75 × 10^6^ km^2^: Parkes et al. 2013). Similar to other radar analysis data, the UK Nimrod radar precipitation analysis has already been bias-corrected (Golding 1998) to remove spurious echoes resulting from anomalous propagation of the radar beam, errors resulting from variations in the vertical profile of reflectivity and radar sensitivity errors. The bias-correction is automatically applied within the Nimrod system, resulting in 30% bias reduction on average (Harrison et al. 2000) in the UK Nimrod radar precipitation analysis. Although there remain considerable errors and uncertainties in the UK Nimrod radar precipitation analysis, it is still particularly useful for capturing the spatial distribution and temporal evolution of precipitation that is of interest in this study.

Over some areas especially oceanic areas where the availability of ground measurements and radar are limited, meteorological satellites provide a unique opportunity for monitoring precipitation. Here, we use the Global Satellite Mapping (GSMaP) hourly precipitation estimate (Ushio et al. 2009) developed by the JAXA Global Rainfall Watch System Earth Observation Research Centre. The GSMaP satellite analysis data integrates retrievals from multi-sensor, i.e. passive microwave (PMW) radiometers and infrared radiation (IR) radiometers. It covers a quasi-global (60° N–60° S) domain with horizontal grid spacing of 0.1° (3600 × 1200 pixels) and one hour intervals, from January 1998 and is updated in real-time.

### Basic theory of OI-based multi-source merging Method

As a powerful method, Optimal Interpolation (OI) is widely used in data merging (e.g., Gandin 1965; Bergman 1978; Daly 1991; Xie and Xiong 2011; Shen et al. 2014; Pan et al. 2015, Shen et al. 2018). Bergman (1978) and Xie and Xiong (2011) given the relatively compact description of the theory of the IO method. The basic idea is to take an irregular distribution of observations of precipitation quality and obtain the best possible estimate of a precipitation field at a regular network of grid points through minimizing the mean square interpolation error for a large ensemble of analysis situations (Bergman 1978).

According to Bergman (1978) and Xie and Xiong (2011), the analyzed precipitation value, $A_{k}$, also called the final merged precipitation value, at a target grid box ($k$) under the OI framework is obtained by adjusting the first guess value ($F_{k}$) at the grid box using observations (i.e. weighted linear sum of the $\widehat{{f_{i} }}$) at and near the target grid box:

1 $$A_{k} = F_{k} + \sum\limits_{i = 1}^{n} {c_{i} } \widehat{{f_{i} }},$$

where $c_{i}$ and $\widehat{{f_{i} }}$ are respectively the weighting and the observed residual at the *i*th grid box (out of n grid boxes) where the observation is located. The “observed residual” $\widehat{{f_{i} }}$ is defined as the precipitation difference between the observation ($O_{i}$) and the first guess ($F_{i}$):

2 $$\widehat{{f_{i} }} = O_{i} - F_{i} ,\;i = 1,2, \ldots ,n,$$

where *n* is the number of observation grid boxes at and near the target grid box within the searching radius. The gauge data interpolated onto the regular network of grid points is usually used as the observation in data merging (e.g., Xie and Xiong 2011; Shen et al. 2014). Key to the OI-based data merging method is therefore to decide the weighting $c_{i}$ at each grid box that has an observation.

Relative to the “true value” of the precipitation ($T$), either the first guess, the observation or the final analyzed value (or merged value) of the precipitation has a difference. Here, we define these errors as:

3 $$\left\{ {\begin{array}{*{20}c} { - f_{i} = F_{i} - T_{i} } \\ {\varepsilon_{i} = O_{i} - T_{i} } \\ {E_{k} = A_{k} - T_{K} } \\ \end{array} } \right.,$$

where $- f_{i}$, $\varepsilon_{i}$ and $E_{k}$ are the “first guess error”, the “observational error” and the “analysis error”, respectively. For convenience of equation derivation, Bergman (1978) defines the opposing value of the “first guess error” as the “true residual” $f_{i}$, i.e.

4 $$f_{i} = T_{i} - F_{i} ,$$

which is used in the following context. According to the definitions of $f_{i}$ and $\varepsilon_{i}$, the “observed residual” $\widehat{{f_{i} }}$ at the *i*th grid box (as shown in Eq. 4) can be written as the sum of the “true residual” $f_{i}$ and the “observational error” $\varepsilon_{i}$:

5 $$\widehat{{f_{i} }} = (T_{i} - F_{i} ) + (O_{i} - T_{i} ) = f_{i} + \varepsilon_{i} .$$

Moreover, the principle of assigning the weighting coefficient $c_{i}$ is thereby to minimize the “analysis error” $E_{k}$ (defined in equation group (6)). In other words, the aim of the OI merging scheme is to make the analyzed (or merged) precipitation field as close as it can be to the “true value” of the precipitation field.

In statistical practice, the $c_{i}$ is chosen so that the mean analysis error ($E_{k}$) variance of a large temporal ensemble, i.e. the $\overline{{E_{k}^{2} }}$, is at a minimum. The $\overline{{E_{k}^{2} }}$ is written as:

6 $$\overline{{E_{k}^{2} }} = \overline{{(T_{k} - A_{k} )^{2} }} = \overline{{\bigg[T_{k} - F_{k} - \sum\limits_{i = 1}^{n} {c_{i} } (f_{i} + \varepsilon_{i} )\bigg]^{2} }} = \overline{{\bigg[f_{k} - \sum\limits_{i = 1}^{n} {c_{i} (f_{i} + \varepsilon_{i} )\bigg]^{2} } }} ,$$

where “$\overline{{\left( {} \right)}}$” denotes the geometric mean over a reference time span. According to the least squares criterion, to minimize the $\overline{{E_{k}^{2} }}$ requires the partial-differentiation of $\overline{{E_{k}^{2} }}$ with respect to each of $c_{i}$ equal to zero, which leads to the equation:

7 $$\sum\limits_{j = 1}^{n} {\overline{{(f_{i} + \varepsilon_{i} )(f_{j} + \varepsilon_{j} )}} } c_{j} = \overline{{f_{k} (f_{i} + \varepsilon_{i} )}} ,\quad i = 1,2, \cdots ,n,$$

We can expand the product terms in Eq. (7) in the following format:

8 $$\sum\limits_{j = 1}^{n} {(\overline{{f_{i} f_{j} }} + \overline{{f_{i} \varepsilon_{j} }} + \overline{{f_{j} \varepsilon_{i} }} + \overline{{\varepsilon_{i} \varepsilon_{j} }} )c_{j} } = \overline{{f_{k} f_{i} }} + \overline{{f_{k} \varepsilon_{i} }} , \quad i = 1,2, \cdots ,n.$$

The linear Eq. (8) may then be solved for the $c_{j}$, provided that the six quantities $\overline{{f_{i} f_{j} }}$, $\overline{{f_{i} \varepsilon_{j} }}$, $\overline{{f_{j} \varepsilon_{i} }}$, $\overline{{\varepsilon_{i} \varepsilon_{j} }}$, $\overline{{f_{k} f_{i} }}$ and $\overline{{f_{k} \varepsilon_{i} }}$ can be specified. Here, we assume that $\overline{{f_{i} }}$ and $\overline{{\varepsilon_{i} }}$ are zero, so that the above six quantities refer to covariance.

In actual computations, it is convenient to express Eq. (8) in a normalized form:

9 $$\sum\limits_{j = 1}^{n} {(\mu_{ij} + \tau_{ij} \sigma_{j} + \tau_{ji} \sigma_{i} + \rho_{ij} \sigma_{i} \sigma_{j} )c_{j}^{\prime } } = \mu_{ki} + \tau_{ki} \sigma_{i} ,\quad i = 1,2, \cdots ,n$$

where

10 $$\left\{ {\begin{array}{*{20}l} {\mu_{ij} = \overline{{f_{i} f_{j} }} /(\overline{{f_{i}^{2} }} \overline{{f_{j}^{2} }} )^{{\frac{1}{2}}} } \\ {\rho_{ij} = \overline{{\varepsilon_{i} \varepsilon_{j} }} /(\overline{{\varepsilon_{i}^{2} }} \overline{{\varepsilon_{j}^{2} }} )^{{\frac{1}{2}}} } \\ {\tau_{ij} = \overline{{f_{i} \varepsilon_{j} }} /(\overline{{f_{i}^{2} }} \overline{{\varepsilon_{j}^{2} }} )^{{\frac{1}{2}}} } \\ \begin{aligned} \sigma_{i} = (\overline{{\varepsilon_{i}^{2} }} /\overline{{f_{i}^{2} }} )^{{\frac{1}{2}}} \hfill \\ c_{j}^{\prime } = (\overline{{f_{i}^{2} }} /\overline{{f_{k}^{2} }} )^{{\frac{1}{2}}} c_{j} \hfill \\ \end{aligned} \\ \end{array} } \right.,$$

Here the $\mu_{ij}$ is the first-guess error correlation at the *i*th and *j*th grid boxes; similarly, the $\mu_{ki}$ at the right-hand side of Eq. (10) is the first-guess error correlation between the *k*th target grid box and the *i*th observation grid box. $\rho_{ij}$ is the observation error correlation at the *i*th and *i*th grid boxes. The $\tau_{ij}$ is the correlation between the first-guess error at the *i*th grid box with the observational error at the *j*th grid box; similarly, the $\tau_{ki}$ at the right-hand side of Eq. (11) is the correlation between the first-guess error at the *k*th target grid box and the observational error at the *i*th observational location. The $\sigma_{i}$ is the ratio of the RMS of the observational error $(\overline{{\varepsilon_{i}^{2} }} )^{{\frac{1}{2}}}$ to the RMS of the first-guess error $(\overline{{f_{i}^{2} }} )^{{\frac{1}{2}}}$ at the *i*th grid box, which is a parameter frequently “tuned” to give more or less weight to the observations (Kalnay 2002), also called the “signal-to-noise ratio” (e.g., Brankart and Brasseur 1996; Barth et al. 2008).

As one observing system is not usually reliant on other observing systems in determining its values, we hypothesise that the random errors in the three input datasets (gauge, radar and satellite) are independent: i.e., the error correlations for the three precipitation analyses are zero, similar to assumptions in previous studies (e.g., Xie and Xiong 2011; Shen et al. 2014; Pan et al. 2012, 2015). Under this hypothesis, no matter which of the three input data is used as the first-guess or observation, the first-guess errors are not correlated with the observational errors, so we get $\tau = 0$ in Eq. (10), and Eq. (11) then simplifies to:

11 $$\sum\limits_{j = 1}^{n} {(\mu_{ij} + \rho_{ij} \sigma_{i} \sigma_{j} )c_{j}^{{\prime }} } = \mu_{ki} .$$

Therefore, through the quantification of errors and error correlations ($\mu_{ij}$, $\mu_{ki}$, $\rho_{ij}$, $\sigma_{i}$, $\sigma_{j}$) in Eq. (12) the weight coefficient $c^{\prime}$ can be solved.

Once the weight coefficients ($c_{i}^{\prime }$) are determined, the analyzed (or merged) value ($A_{k}$) can then be defined from the first guess and the observations according to Eqs. (10) and (1), as follows:

12 $$\frac{{A_{k} - F_{k} }}{{(\overline{{f_{k}^{2} }} )^{{\frac{1}{2}}} }} = \sum\limits_{i = 1}^{n} {c_{i}^{{\prime }} \frac{{(O_{i} - F_{i} )}}{{(\overline{{f_{i}^{2} }} )^{{\frac{1}{2}}} }}} .$$

Here $(\overline{{f_{k}^{2} }} )^{{\frac{1}{2}}}$ and $(\overline{{f_{i}^{2} }} )^{{\frac{1}{2}}}$ are the RMS of the first-guess error at the *k*th target grid box and at the *i*th observation grid box, respectively.

Based on the theory of OI presented above, we know that key to the development of an OI–based combined algorithm is the quantification of errors and error correlations for the input first guess and the observations. Specifically, there is an important assumption in OI theory that $\overline{{f_{i} }}$ and $\overline{{\varepsilon_{i} }}$ are zero. This is equivalent to the assumption that both the first guess and observation values contain no systematic errors. The removal of the systematic errors thereby becomes an important step in our data merging scheme as shown in the next section.

### Evaluation metrics for data comparison

Data errors consist of systematic errors and random errors (Taylor 1997). The systematic errors (also called bias errors) are consistent, repeatable errors, which usually come from the measuring instruments. The random errors are unrepeatable, inconsistent errors in the measurements, which come from random causes and the average of these random errors is usually zero. In practice, for data comparison, the gauge data (observations or gauge analysis) is usually used as the “ground truth” for the precipitation (e.g., Xie and Xiong 2011; Shen et al. 2014), as its value is much closer to the “true value” of precipitation relative to other indirect measurements such as radar and satellite data. Depending upon the context, the “ground truth” precipitation can be defined as: (1) the individual gauge observation values located in the gauge network; (2) the arithmetic mean values of gauge observations within the grid boxes; or (3) the gauge interpolation analysis values at the grid boxes with at least one reporting gauge, which will be specified in the study.

To measure the errors of the precipitation analysis data (the pre-processed input data and the merged data) against the “ground truth” precipitation, we employ four commonly used (e.g., Xie and Xiong 2011; Shen et al. 2014) statistical metrics in this study: (1) bias, (2) root-mean-square error (RMSE), (3) error variance and (4) correlation coefficient (CC).

1. Bias is defined as:
  13 $$Bias = \frac{1}{n}\sum\limits_{i = 1}^{n} {(p_{i} - g_{i} )}$$
  where $p_{i}$ is the precipitation value of the target data to be assessed (i.e. the pre-processed input data or the merged data), and $g_{i}$ is the “ground truth” precipitation, usually represented by gauge data or gauge analysis data; and $n$ is the number of samples. The Bias is the mean difference between the target precipitation field and the precipitation “ground truth” field. As the average of the random errors is zero, the Bias metric actually represents the systematic errors. Bias ranges from negative infinity to positive infinity. A zero value for Bias represents no systematic errors in the target data (i.e. the pre-processed input data or the merged data) against the “true value” of precipitation.
2. RMSE is defined as:
  14 $$RMSE = \sqrt {\frac{1}{n}\sum\limits_{i = 1}^{n} {(p_{i} - g_{i} )^{2} } } .$$
  RMSE measures the magnitude of overall errors in the target precipitation data, including both the random errors and the systematic errors. RMSE ranges from zero to infinity, with the value of zero representing the best accuracy of the target precipitation data (or the least overall error magnitude against the “ground truth” precipitation).
3. Error Variance is defined as the squared value of the RMSE.
4. CC is defined as:
  15 $$CC = \frac{{\sum\nolimits_{i = 1}^{n} {(p_{i} - \bar{p})(g_{i} - \bar{g})} }}{{\sqrt {\sum\nolimits_{i = 1}^{n} {(p_{i} - \bar{p})^{2} } } \sqrt {\sum\nolimits_{i = 1}^{n} {(g_{i} - \bar{g})^{2} } } }},$$
  where $\bar{p}$ and $\bar{g}$ are respectively the average values of $p_{i}$ and $g_{i}$. CC can show the similarity degree of the target data with the “ground truth” precipitation in either the temporal or the spatial dimension. CC ranges from − 1 to 1. CC equal to one represents the best agreement of the target precipitation data with the “ground truth”: for the spatial dimension at one time step, it suggests the matching of spatial patterns; for the temporal dimension at one spatial location, it suggests the matching of temporal variability.

In addition, the RMSE and the CC can also be used to assess the differences between any other two fields. A bigger value of RMSE or smaller value of CC indicates a bigger difference between the two fields, and vice versa.

### Methods of independent tests for the experimental UKGrsHP

To evaluate the performance of the merging results, we use an independent test. Prior to data merging, we remove 407 gauges (Fig. 1b) from MIDAS, which does not take part in the merging process. These are from an independent network and are then used as independent gauges to evaluate the precipitation estimates from the merged product. MIDAS is one out of four original gauge data sources (as detailed in Sect. 2.1)—MIDAS, EA, NRW and SEPA. The spatial distribution of the MIDAS gauges is relatively homogeneous over the UK as evidenced in Fig. 1b, in contrast, the distributions of the gauges compiled from other sources [EA, NRW and SEPA, as shown in Fig. 1 of Lewis et al. (2018)] are separately located only over parts of UK, i.e. either England, Wales, or Scotland. The 1496 remaining gauges from EA, NRW and SEPA (shown as spatial distribution in Fig. 1c, and red line in Fig. 1d shows the actual number participated in data merging from hour to hour) are used in the merging process.

To avoid one or more reporting gauges (in experimental data merging) being available in the same grid boxes of the independent stations that may influence the justice of the independent test, we removed any MIDAS station within 1 km of any merging station. On average, about 30 MIDAS gauges are removed before the independent tests. Therefore, around 220 MIDAS gauges are used in the independent test from hour to hour (blue line in Fig. 1d).

The inverse distance weighting (IDW) method (Simanton and Osborn 1980) is used in the independent check, in which the target precipitation data to be assessed (the experimental UKGrsHP and the input precipitation analysis data) are interpolated back onto the locations of the ~ 220 independent check stations. These are then compared with the “ground truth” precipitation represented by the independent check gauges based on the RMSE and the CC, as defined in Sect. 2.2.

## The flowchart of the data merging steps of UKGrsHP

To begin with, we introduce the flowchart of our data merging scheme in this section. To merge the three data sources (gauge, satellite and radar), we pre-process them onto the same horizontal resolution grid boxes before the merging. Then we: (1) remove the systematic bias in the radar and satellite estimates through their comparison/calibration against gauge observations, and; (2) further combine the gauge analysis with the bias–corrected radar estimates and the bias–corrected satellite estimates to reduce the random errors. The above merging steps developed in this study are based on the conceptual model of Xie and Xiong (2011).

**Fig. 2:**
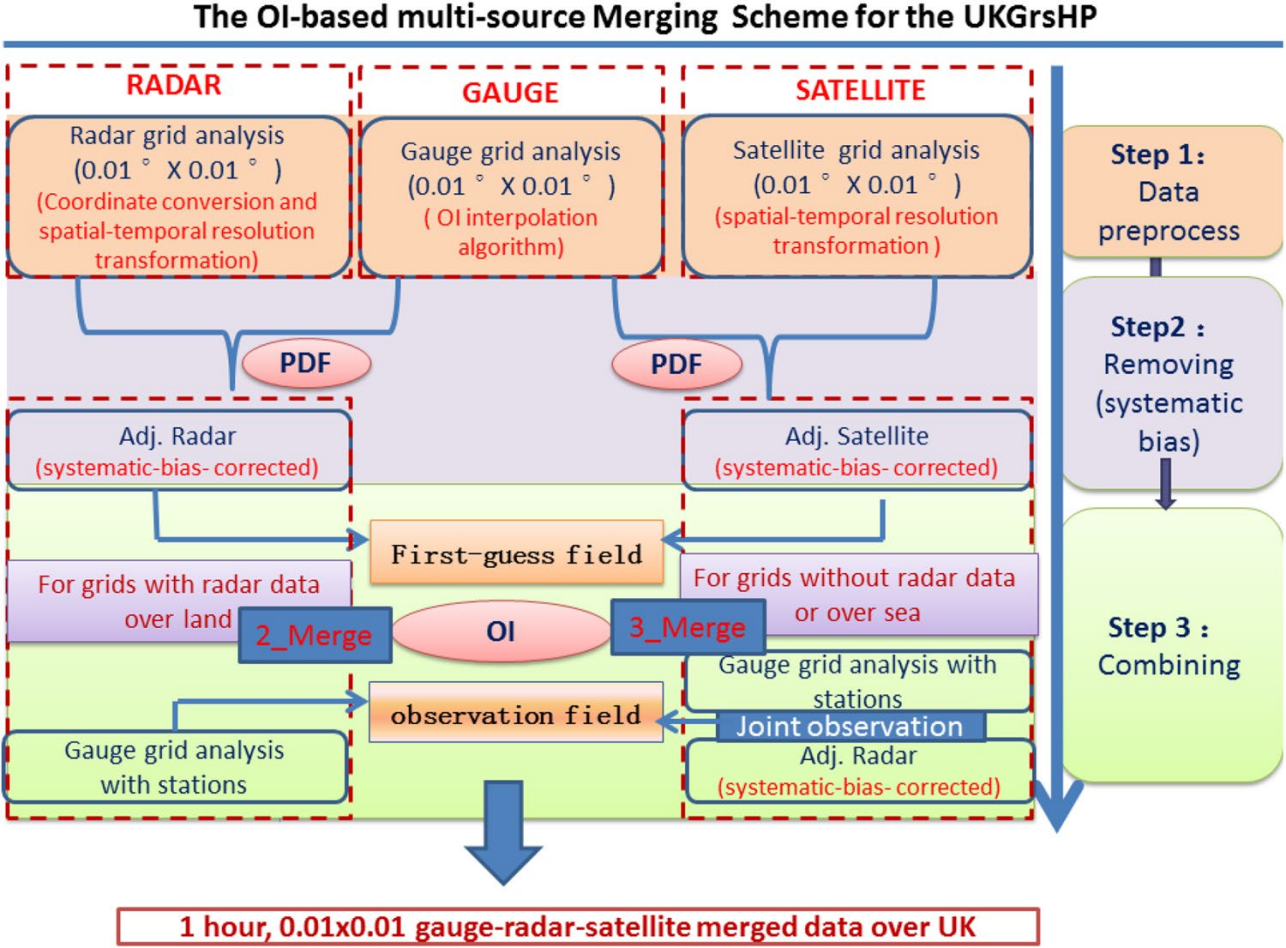
The flowchart of the OI-based multi-source Merging Scheme for the UKGrsHP. The terms ‘GAUGE’, ‘SATELLITE’ and ‘RADAR’ respectively represent the gauge precipitation observations, the GSMaP satellite precipitation analysis and the Nimrod radar analysis. The methods ‘PDF’ and ‘OI’ respectively refer to the probability density function matching method and the Optimal Interpolation merging method. The terms ‘Adj. radar’ and ‘Adj. Satellite’ respectively denote the bias-corrected radar analysis and satellite precipitation estimates by PDF. The ‘2_Merge’ and ‘3_Merge’ represent the two different merging algorithms as described in Sect. 6.4

In short, there are three main steps to the data merging process in the OI-based multi-source Merging Scheme for the UKGrsHP. We summarize the major steps of the merging scheme in the flowchart of Fig. 2 as:

1. pre-processing of the gauge, radar and satellite precipitation data,
2. systematic bias removal from the pre-processed radar and satellite data by using the Probability Density Function (PDF) method (e.g., Yu et al. 2013), and
3. combining the data based on the OI method.

The processes and analyse results for each step are detailed in the following sections.

## Step 1: data pre-processing

**Fig. 3:**
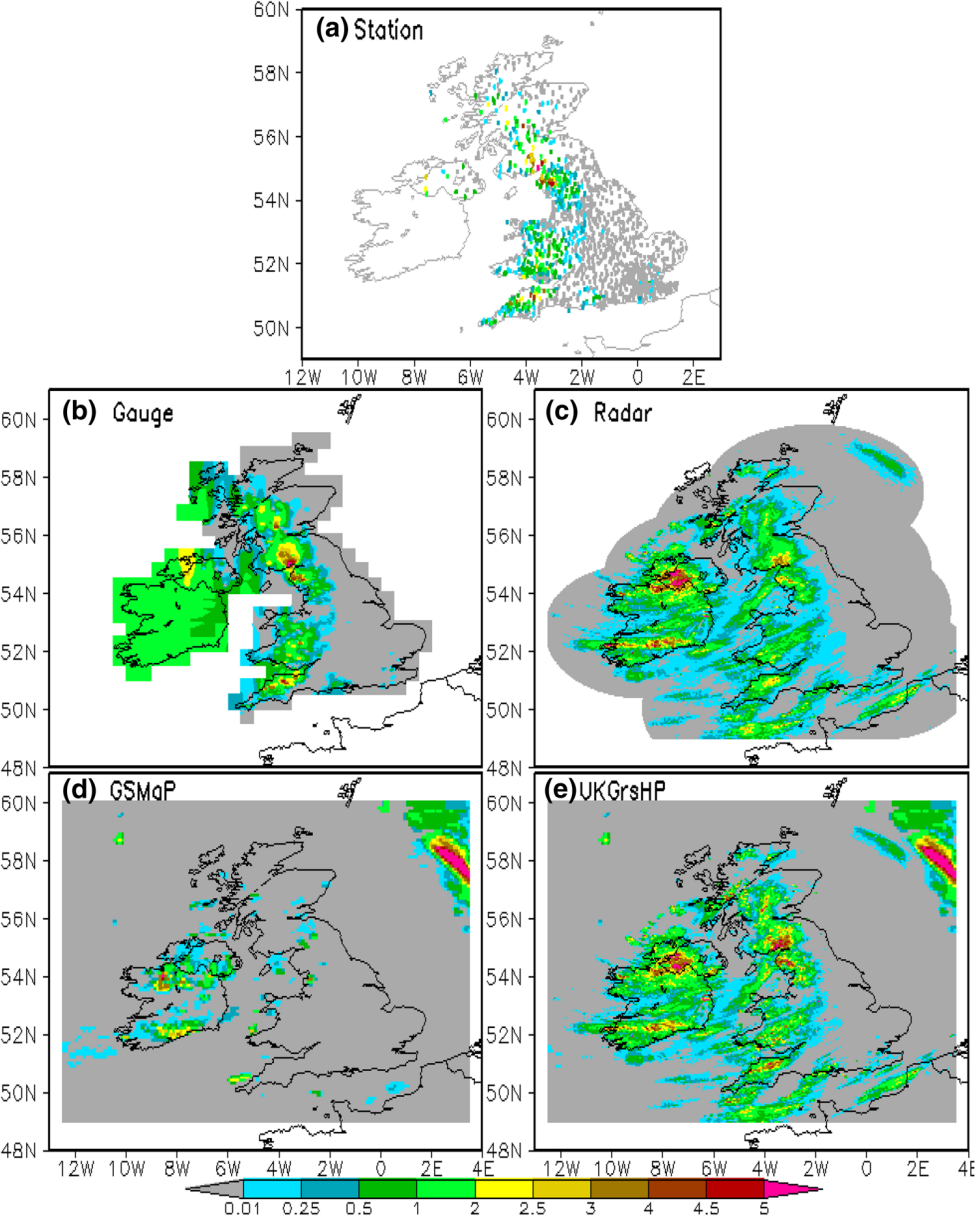
Spatial distribution of hourly precipitation (mm/h) at 12Z, 1st February 2014. **a** Station observations, **b** gauge analysis data interpolated from 1903 rain station observations, **c** Nimrod radar analysis data, **d** GSMaP satellite analysis data, and **e** the merged product, i.e. the experimental UKGrsHP. The experimental UKGrsHP is merged from the 1903 gauge observations, the Nimrod radar analysis and the GSMaP satellite analysis

Before merging, the three input precipitation datasets from gauge, satellite and radar are pre-processed to bring them onto the hourly time scale and the same regular grid. The target regular grids are for the wider UK area of 12.5° W–3.5° E, 49° N–60° N, with the interval of 0.01º in both latitude and longitude (equivalent to 1 km × 1 km in the mid-latitudes). The data merge results, i.e. the experimental UKGrsHP dataset, are also for the same area with the same spatial resolution. After data pre-processing, the three input datasets are referred to as gauge analysis, satellite analysis and radar analysis, respectively. Here, we provide five precipitation events randomly chosen across different seasons in 2014, which are presented in three pre-processed analysis data as examples of the pre-processing results. In the main text, we only use the precipitation events at 12Z on 1st February (Fig. 3) as an example, the other four events can be examined in Sect. 1 of the Supplementary Information.

### Rain gauges

The distribution of high-resolution rain gauges over the UK is heterogeneous and sparse relative to the target 0.01° resolution. Figure 1a shows that there is a relatively more dense distribution of rain gauges over the southern UK, with sparser distribution in northern regions. Even over southern England most grid boxes with 0.01° resolution contain no rain gauges. The heterogeneous and sparse distribution of rainfall gauges relative to the 0.01° resolution grid is one important reason for us to develop an hourly merged precipitation dataset, which can extract useful information from radar and satellite data to enhance the quality of the precipitation data, especially in areas without rain gauges.

In pre-processing, by use of a modified climatology-based OI interpolation algorithm (e.g., Xie et al. 2007; Shen et al. 2010a, b), we interpolate the 1903 hourly quality-controlled gauge data onto the regular grid points with a spatial resolution of 0.01° over the mainland UK. The interpolation algorithm (e.g., Xie et al. 2007; Shen et al. 2010a, b) is better than normal interpolation methods in preserving inhomogeneous features of the spatial distribution of hourly precipitation. Figure 3b shows an example of gauge data for 1st February 2014 at 12 Z (hourly rainfall accumulation from 12 UTC to 13 UTC), which is interpolated into the gauge-based hourly precipitation interpolation data (Fig. 3b, hereafter called gauge analysis data). From Fig. 3b, we can see the gauge analysis data performs well in representing the intense rainfall signal over the UK, as evidenced by a series of heavy rainfall areas distributed along the western part of the UK which has a denser distribution of gauges (Fig. 3a), especially along a latitudinal belt near 55° N where the maximum rainfall rate exceeds 5 mm/h. However, the gauge analysis data obviously lacks precipitation information on the rainfall centred over Ireland (Fig. 3b). This is mainly due to limited availability of gauge observations over Northern Ireland and the lack of gauge observations available to this study over Ireland (Fig. 3a). In contrast, both remote sensing analyses, the radar analysis (Fig. 3c) and the satellite analysis (Fig. 3d), provide precipitation information over Ireland. Moreover, interpolation methods (including the modified climatology-based OI interpolation algorithm itself) tend to produce a smooth spatial field between gauge observations, which are different to the real spatial distribution of hourly rainfall intensities; the latter usually being highly inhomogeneous and not smoothed (e.g., Xie et al. 2007). As a result, gauge analysis data based on the interpolation of sparse gauges has limitations in giving a reliable and detailed spatial estimation of hourly rainfall, especially over areas without rain gauges.

### Radar-based quantitative precipitation estimation (QPE)

In pre-processing, we accumulate the Nimrod radar data from 5 min to 1 h temporal intervals. If one or more snapshots of 5 min precipitation rate are unavailable for a given hour, the precipitation rate for that hour is marked as missing. Then, using the IDW method, we interpolate the hourly Nimrod radar data from a 1 km × 1 km resolution on the British National Grid (BNG) onto a horizontal resolution of 0.01° × 0.01° in latitude and longitude in the geographical coordinate system. Figure 3c presents an example of the hourly radar data with a resolution of 0.01° (hereafter called radar analysis data) at 12 Z on 1st February 2014. Comparing Fig. 3b with Fig. 3c, we can see the radar analysis data (Fig. 3c) provides more spatial rainfall detail than the gauge analysis data (Fig. 3b). However, we also notice that the radar analysis data (Fig. 3c) obviously underestimates heavy hourly precipitation over the UK compared to the gauge analysis data (Fig. 3c). Thus, the radar analysis data provides better spatial pattern information, but weaker rainfall amount information.

### Satellite-based precipitation product

To pre-process the hourly GSMaP precipitation data, we directly downscale it from a spatial resolution of 0.1° × 0.1° to 0.01° × 0.01° in the target area, with the same precipitation value assigned to all 0.01° × 0.01° grid cells falling within a 0.1° × 0.1° grid cell, and hearfter called satellite analysis data. As shown in Fig. 3d, the satellite analysis data has a broader spatial coverage than both the gauge analysis data and the radar analysis data, and covers ocean areas adjacent to the mainland UK, which are not covered well by either the gauge or radar data. However, the satellite analysis data has limitations in depicting the features of precipitation over mainland UK, which is a common issue for satellite precipitation datasets over land areas in the mid-to-high latitudes (e.g. Serreze et al. 2005; Cai et al. 2015; Sun et al. 2018). As evidenced in Fig. 3d, the satellite analysis data only depicts the precipitation centre over Ireland, but misses the precipitation centres over western Britain that are depicted well in both the gauge analysis data (Fig. 3b) and the radar analysis data (Fig. 3c). Misrepresentation of land precipitation spatial patterns or large errors of satellite precipitation over land areas are commonly found in satellite analysis data.

## Step 2: PDF systematic-bias-correction of radar analysis and satellite analysis data

We adopt the probability density function matching method (PDF) to adjust the systematic bias of the pre-processed radar and satellite precipitation data. Systematic errors of remote sensing precipitation data, such as radar and satellite, usually change temporally and spatially; called range-dependent biases (e.g., Chiang et al. 2007; Hong et al. 2007; Tian et al. 2009; Xu and Xie 2010; Shen et al. 2018). The PDF systematic-bias-correction method has been demonstrated as an effective way in reducing the range-dependent biases in remote sensing precipitation data (e.g., Xie and Xiong 2011; Yu et al. 2013; Shen et al. 2014; Li et al. 2015). The steps of the PDF systematic-bias-correction are to: (1) collect co-located pairs of gauge and radar (satellite) analysis data over the 0.01° lat/lon grid boxes with ≥ 1 reporting gauges within a spatial window centred on the target grid box, and within a temporal window ending at the target date; (2) match the value of cumulative PDFs (CPDFs) of the radar (satellite) analysis data against those of the gauge analysis data to remove the bias, assuming the CPDFs of the gauge analysis data represent the CPDFs of the ground truth precipitation. Considering the highly discontinuous nature in the temporal dimension of UK hourly precipitation, the temporal window in our strategy of the PDF matching is only selected at the target hour, i.e. 1 h of temporal window. Here, more than 150 non-zero pairs of data samples are collected to ensure stable statistics of the CPDFs, so the spatial domain centred at the target grid box may need to be expanded wherever necessary, especially over gauge-sparse areas.

**Fig. 4:**
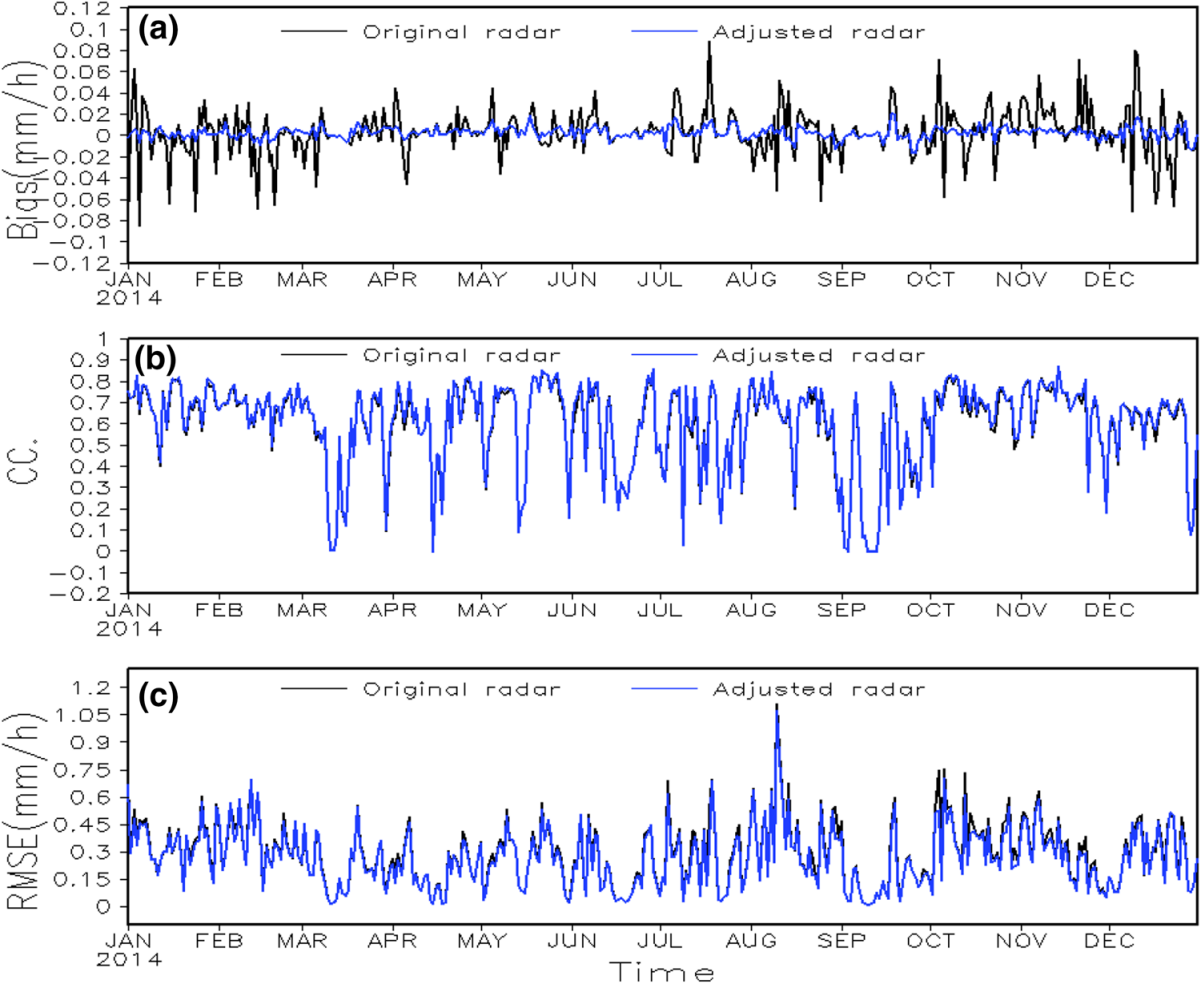
Time series of the data errors of the original (black) and PDF-adjusted (blue) radar analyses. The data errors are measured by the metrics of the daily mean **a** Bias (mm/h), **b** CC and **c** RMSE (mm/h), which are calculated against the “ground truth” represented by the hourly gauge analysis data over grid boxes with at least one reporting gauge observation

To evaluate the effectiveness of the PDF systematic-bias-correction method, the original and the adjusted data are compared with the “ground truth” by using three statistical metrics: Bias, CC and RMSE (Fig. 4). The “ground truth” here is represented by the value of the gauge analysis data at all grid boxes with at least one reporting gauge.

Figure 4a shows that the systematic data errors are decreased remarkably by the PDF systematic-bias-correction. The range of Bias values (representing the systematic data errors, more details in Sect. 2.3) are close to zero after applying the PDF systematic-bias-correction method (blue curve in Fig. 4a), in contrast to the much larger Bias of the initial radar data (black curve in Fig. 4a). Moreover, the CC (Fig. 4b) changes very little after the PDF systematic-bias-correction (blue curve vs black curve in Fig. 4b), suggesting the PDF systematic-bias-correction method does not destroy the basic spatial structure of the radar data. However, the mean RMSE is only decreased slightly by the correction: from 0.284 to 0.269. Given that the RMSE represents the magnitude of the total data errors, including both the systematic and the random data errors, and systematic data errors are significantly removed, the slightly changed RMSE implies that there are still large random data errors in the radar data. Therefore, while the PDF systematic-bias-correction removes most of the systematic data errors in the radar data, the random data errors still need further processing.

We also apply the PDF systematic-bias-correction method to the pre-processed satellite data. The effect of the PDF systematic-bias-correction on the satellite analysis data is lower due to more zero values in the satellite analysis data than the radar analysis data, but we still get similar results (figures not shown). The systematic data errors in the satellite analysis data are removed well with only a small change in basic spatial structure, but the random data errors are still considerable.

## Step 3: OI-based multi-source merging for the UKGrsHP

After the pre-processing and systematic-error removal steps, we now merge the three input data sources (the gauge analysis, the systematic-bias-corrected satellite analysis and the systematic-bias-corrected radar analysis) using the OI method. The key to developing an OI-based multi-source merging scheme is to quantify the errors and error correlations ($\mu_{ij}$, $\mu_{ki}$, $\rho_{ij}$, $\sigma_{i}$, $\sigma_{j}$) in the first-guess and the observation fields, as denoted in Eq. (12). Moreover, as we have three input data sources, the merging strategy (or how to assign the first-guess and the observation fields under the OI scheme) is also important for the quality of the merging result. In this step, we thereby investigate the statistical features of the error structure in the individual input analysis data though limited samples, then estimate the errors and error correlations based on the empirical equations derived from them to decide on the final merging strategy.

### Errors in data sources

The random error in the three unbiased precipitation analysis data at a grid box is defined as the difference of the precipitation analysis value from the “ground truth” represented by the arithmetic mean of high-density gauge values within the 0.01º latitude/longitude grid box [also see Eq. (3)]. The basic features of the random error in the three precipitation analysis datasets is essentially depicted (Xie and Xiong 2011; Shen et al. 2014, 2018) by changes of the error magnitudes with both the precipitation intensity and the sample size. As mentioned before, the density of hourly gauge observations over the UK is sparse and spatially varying. In fact, the number of reported gauge observations within most 0.01° × 0.01° grid boxes over the UK is less than 2; too small to examine the change in the error magnitude with sample size. So, we mainly quantify the change of error magnitude against precipitation rate.

**Fig. 5:**
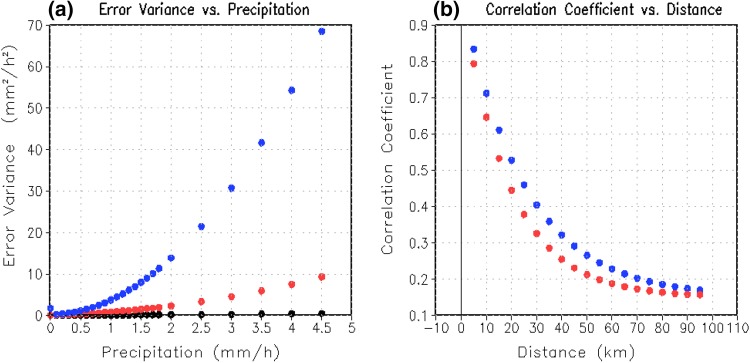
**a** The error variance of the hourly gauge analysis data (over grid boxes with at least one recording gauge, black dots), radar analysis data (red dots) and satellite analysis data (blue dots) as a function of rainfall intensity, and **b** error correlation of radar (red dots) and satellite (blue dots) analysis data with distance between any two points as an exponential function

In practice, we use the Error Variance (see metric definition in Sect. 2.2) to represent the magnitude of the errors. It is calculated at grid boxes where there is at least one reporting gauge observation during 2014. The error variance and the arithmetic mean precipitation rate are calculated for precipitation rate bins starting from 0 mm h^−1^ with an increment of 0.1 mm h^−1^. The approximate binomial polynomial fitting (Storch and Zwiers 1999) curves of the scatter pairs of the Error Variance and the corresponding mean precipitation rates within different precipitation rate bins is calculated and plotted in Fig. 5a. Figure 5a indicates that one common feature of data errors in all three precipitation analysis datasets is that the random errors are proportional to the precipitation intensity: all three Error Variance fitting curves increase non-linearly with increase in precipitation rate. This is similar to results from previous studies (e.g., Huffman, 1997; Li et al. 1998; Bell and Kundu 2000). Moreover, we find the magnitude of Error Variance in the systematic-bias-corrected satellite analysis (blue dot line in Fig. 5a) is much bigger than that for either the gauge analysis data (black dot line in Fig. 5a) or the systematic-bias-corrected radar analysis data (red dot line in Fig. 5a). In particular, the magnitude of the Error Variance in the systematic-bias-corrected satellite analysis is nearly six times larger than that in the systematic-bias-corrected radar analysis data for heavy rainfall. In addition, the gauge analysis data (black dot line in Fig. 5a) has the least magnitude of error variance, which is reasonable as the Error Variance is calculated at grid boxes where there is at least one reporting gauge. Therefore, the bigger the hourly precipitation intensity, the larger the magnitude of the random errors in all three precipitation analyses.

### Error correlation in data sources

The error correlations in the systematic-bias-corrected satellite analysis or systematic-bias-corrected radar analysis data are calculated between pairs of grid boxes that contain at least one reporting gauge observation. For the gauge analysis data, the error correlations between two different locations are very small and usually assumed as zero (e.g., Xie and Xiong 2011; Shen et al. 2014), as the observational error over two gauges tend to be independent to each other. For the satellite and radar analysis data, error correlations usually decrease exponentially with distance (e.g., Xie and Xiong 2011; Shen et al. 2014; Pan et al. 2015). So we hypothesise that: (1) error correlations in the gauge analysis between two different locations are zero; (2) in the other two analysis data, the error correlation is a function of the distance between the two points, expressed as an exponential function of the negative distance.

Here, we calculate the error correlations between any two different grid boxes where there is at least one reporting gauge observation, then average the error correlations within different groups according to distance between the two grid boxes. The least square fitting curves of the scatter pairs of the averaged error correlations and the corresponding distances is calculated. As shown in Fig. 5b, the error correlations ($r$) in both the systematic-bias-corrected satellite analysis data and systematic-bias-corrected radar analysis data decrease sharply as an exponential function of the distance ($h$): $r = e_{r} \times e^{{ - h/h_{r} }} + c_{r}$ (Xie and Xiong 2011), where $h_{r}$ is the e-folding distance, and $e_{r}$ and $c_{r}$ are constants. In our case, for the hourly satellite analysis and radar analysis data over a 0.01° latitude/longitude grid box, the e-folding distances ($h_{r}$) are respectively set to 25 km and 20 km based on an inspection of the scatterplots (Fig. 5b).

### Three-source and two-source merging methods

The basic OI method needs only two input data fields: the observation field (O) and the first-guess (F) field. Here, we have three input datasets: the gauge, radar and satellite analysis data. As the gauge analysis data has the highest accuracy among the three input data (black dot line in Fig. 5a), this is used as the observation field. How to assign the other two analysis data is important for setting the OI-based merging scheme. Here, we propose two different merging frameworks:

1. In the first merging framework (noted as 3_Merge), we use both the gauge analysis data and the radar analysis data as the observation field. It should be noted that, for the gauge analysis data, only values at grid boxes with at least one reporting gauge observation are used. We use the bias-corrected satellite analysis as the first-guess field. So information from all three input analysis data are used in the merging.
2. In the second merging framework (noted as 2_Merge), we exclude the use of the satellite analysis data as it is demonstrated to contain the biggest errors among the three input datasets. Instead, we use the gauge analysis data at grid boxes with at least one reporting gauge observation as the observation field, and use the bias-corrected radar analysis as the first-guess field. So only two input datasets are used in this merging scheme.

Under the first merging framework (3_Merge), we can now specify the errors and error correlations in Eq. (11). The first-guess error coefficient, $\mu$, is calculated using the satellite analysis data. Given that we have estimated the error correlation between grid boxes for the gauge and radar analysis data, we can now define the observation error correlation,$\rho$, as the following:

$$\left\{ {\begin{array}{*{20}l} {i{\text{th}},j{\text{th are both for gauge,}}} \hfill & {\rho_{ij} = \;\left\langle {\begin{array}{*{20}l} {1\, (i = j)} \\ {0\, (i \ne j)} \\ \end{array} } \right.} \hfill \\ {i{\text{th}},j{\text{th are both for radar,}}} \hfill & {\rho_{ij} = \left\langle {\begin{array}{*{20}l} {1\, { (}i = j ) { }} \\ {e_{r} \times e^{{ - dist(i,j)/h_{r} }} + c_{r} \, (i \ne j)} \\ \end{array} } \right.} \hfill \\ {i{\text{th}},j{\text{th are for radar and gauge, resepectively,}}} \hfill & {\rho_{ij} = 0} \hfill \\ \end{array} } \right.$$

where $i,j = 1,2, \ldots ,n$. The observation field sample size, $n$, is the sum number of grid boxes of both the radar analysis and gauge analysis data (only grid boxes with gauge observation(s) are counted) at and near a target grid box (*k*) within the analysis radius.

Under the second merging framework (2_Merge), the bias-corrected radar analysis data is used as the first-guess field, and the gauge analysis data at grid boxes with at least one gauge observation is used as the observation field. So, the error correlation,$\mu$, is calculated by using the radar analysis data; hence, the observation error correlation, $\rho$, can be simplified to:

$$\rho_{ij} = \left\{ {\begin{array}{*{20}l} {1 \, (i = j)} \\ { 0 \,{ }(i \ne j)} \\ \end{array} } \right.,$$

where $i = 1,2, \ldots ,n$, and $n$ is simply the number of grid boxes where gauge observations are available within the analysis radius.

In either of the above merging frameworks, the weight coefficient, $c$, can be solved using Eq. (11) according to the above specified errors and error correlations, resulting in the final solution of the analyzed (or merged) precipitation according to Eq. (11).

**Fig. 6:**
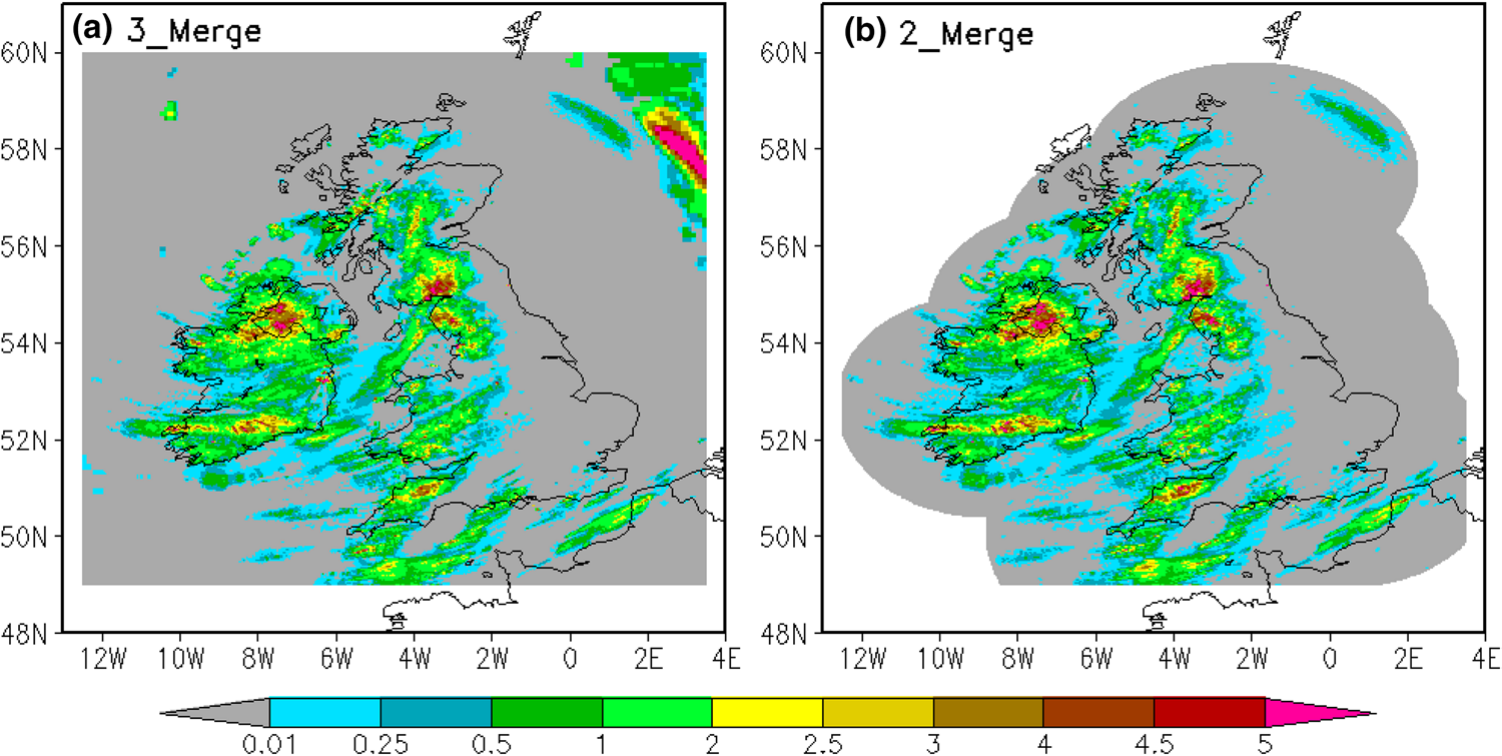
Spatial distribution of hourly merged precipitation (mm/h) using **a** 3_Merge and **b** 2_Merge framework at 12Z, 1st February 2014

An example of the merged hourly data at 12Z on 1st February 2014, produced by the two merging frameworks is presented in Fig. 6. Here, all the available gauge observations are used in the data merging. Comparing Fig. 6a, b, we can see that the merged precipitation from the first scheme (3_Merge) provides better spatial coverage than the second scheme (2_Merge). This is because the satellite analysis data merged by the first scheme (3_Merge) provides more information over oceanic areas, which lack precipitation estimates from either gauge or radar analysis data. However, over land and near-shore areas, which the radar analysis data covers, the merged precipitation from the two merging frameworks are generally similar to each other.

Four other examples of merged hourly data randomly chosen for different seasons produced by the two merging frameworks are presented in Figures S5 to S8 in Sect. 2 of the Supplementary Information. All four examples show that the merged precipitation from the two merging frameworks are generally similar to each other over land areas.

**Fig. 7:**
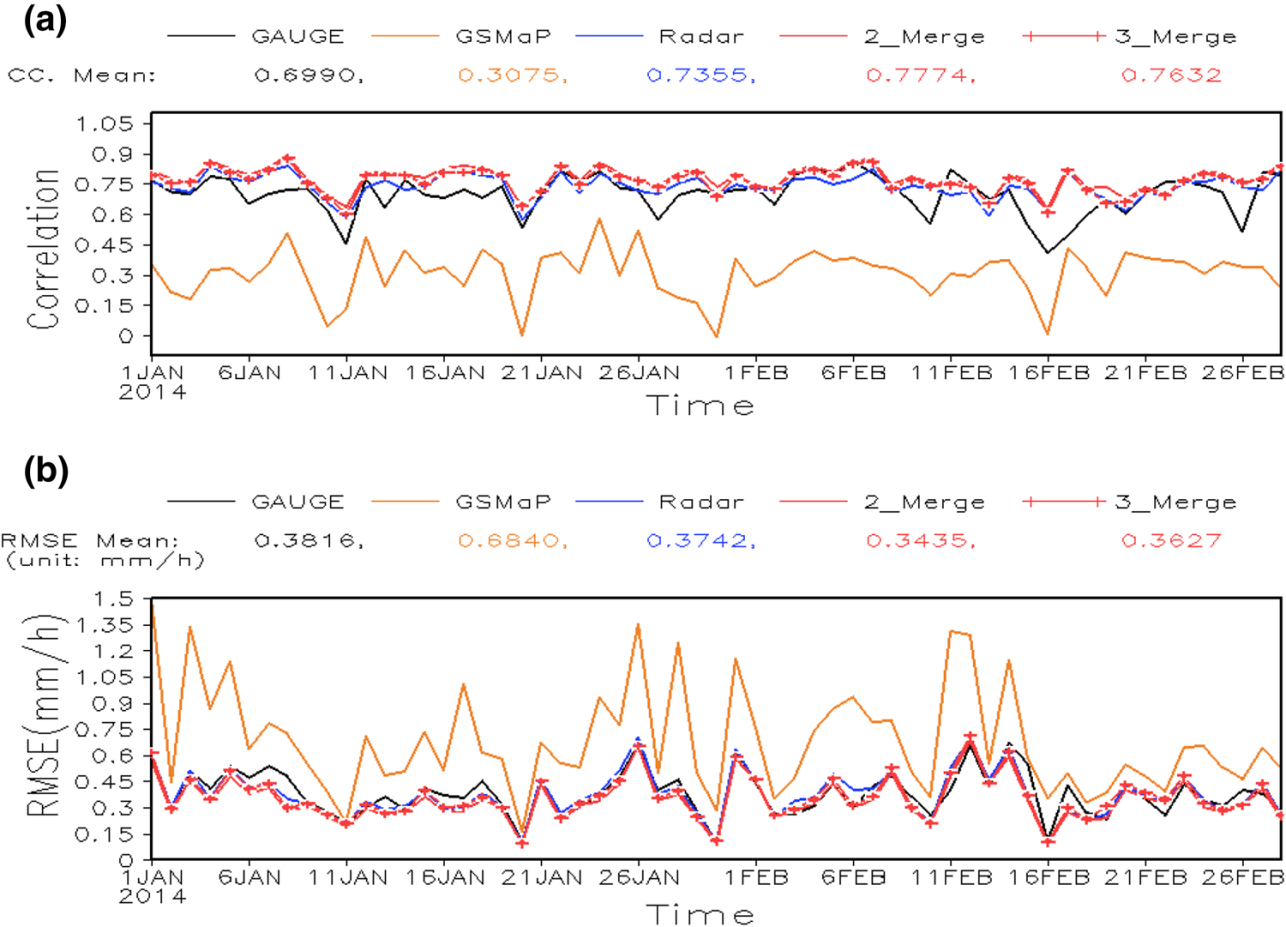
Time series of daily data errors represented by **a** CC and **b** RMSE (mm/h) of 3_Merge (red line with plus sign), 2_Merge (red line), radar (blue), satellite (orange) and gauge (black) analysis data from January–February 2014 for the independent test. The time mean values of CC and RMSE respectively for each analysis are shown in the legends

To further verify the similarity over UK land areas between the merged data produced by the two merging schemes, we now quantify their errors independently. Two month’s independent test results produced by the two merging schemes over UK land areas are used, spanning January–February 2014. The independent test gives objective and more stringent measurements of the performance of the merging results, as the merging results are directly compared with independent gauge observations that are not used in the data merging (more details are described in Sect. 2.3). Two metrics (CC and RMSE) are used to quantify the data errors of the two merging results against the “ground truth” represented by ~ 220 independent gauge observations. Shown as Fig. 7, the errors in the merged data of the 3_Merge scheme (red line with plus sign) and 2_Merge scheme (red line) are similar to each other, with similar values for CC (Fig. 7a) and RMSE (Fig. 7b). In addition, we emphasize that the merging result from either scheme has a better accuracy than all three individual input datasets, evidenced by the lower CC and higher RMSE of satellite (orange line), radar (blue line) and gauge (black line) analysis data than for the merging results from either scheme.

In summary, the merged precipitation data from the first merging framework (3_Merge) has better spatial coverage over ocean areas, but over land and near-shore where radar and gauge analysis data is available, the merged results of the two schemes are similar. Therefore, the satellite analysis used in the first framework (3_Merge) does not significantly improve the performance of the merging results over these areas but requires three times the computational resource.

### Compound merging strategy and its verification

Therefore, to improve computational efficiency we use a compound merging strategy in our OI–based multi-source merging scheme; employing both the 3_merge and 2_merge schemes in the data merging. As the analyzed (or merged) precipitation value is calculated grid box by grid box, the choice of merging scheme is different at each grid box according to whether there are radar analysis data available over land areas. Figure 2 shows a schematic of the merging framework. If the radar analysis value is available at the target grid box over land areas and at least one gauge data can be found at and near the target grid box within the searching radius, then we use the 2_Merge scheme, which assigns the bias-corrected radar analysis as the first-guess field at the target grid box, and assigns the gauge analysis values within the search scope as the observation field to modify the first-guess field value at the target grid box. Otherwise, if there is no radar analysis value available at the target grid box or no gauge data at and near the target grid box within the searching radius, or over sea, we use the 3_Merge framework, which assigns the bias-corrected satellite analysis as the first-guess field at the target grid box, and assigns the radar and gauge analysis within the search scope as the joint observation field. By using the above compound merging strategy, the OI–based multi-source merging scheme (Fig. 2) is built up.

**Fig. 8:**
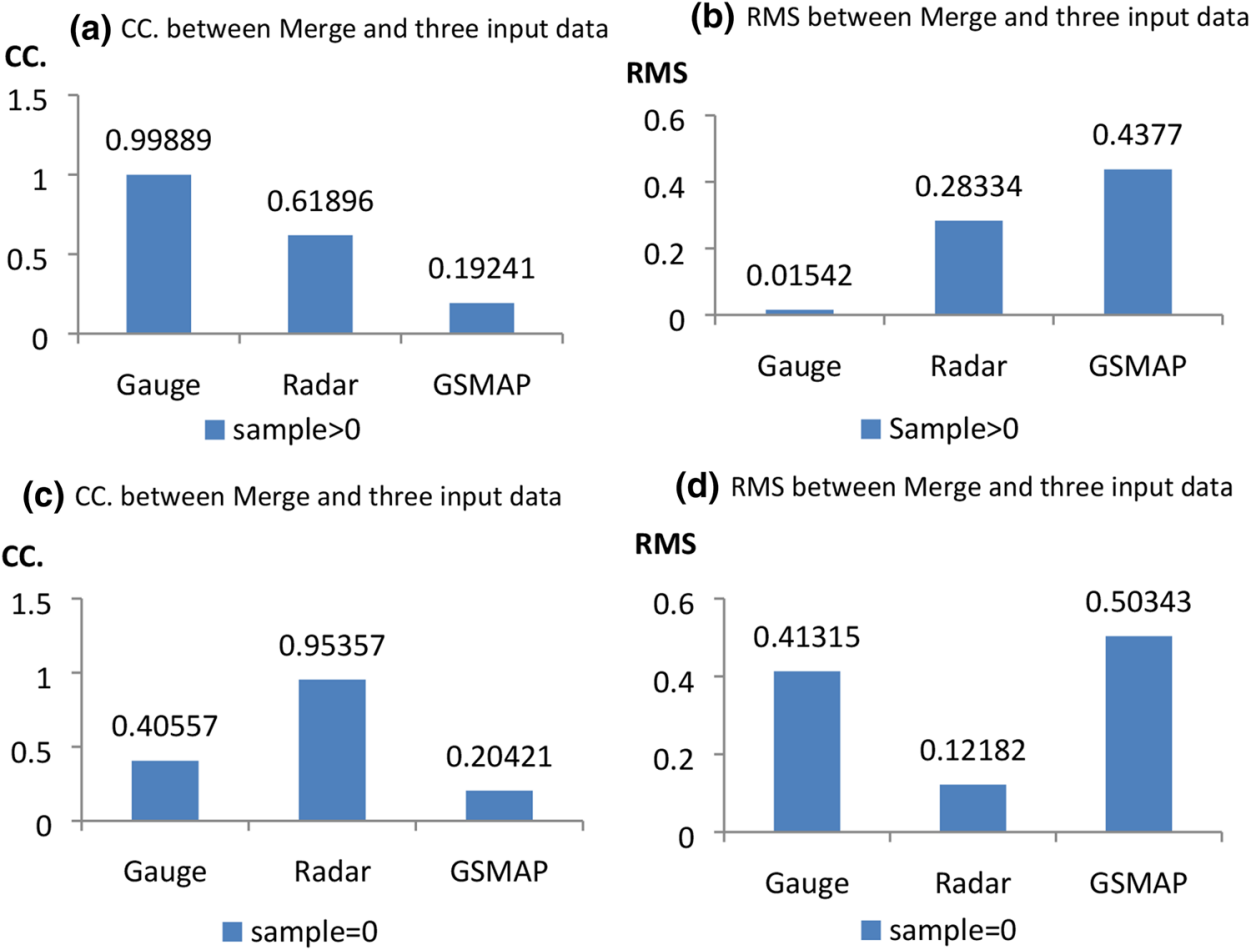
Annual mean data errors for the experimental UKGrsHP and three input precipitation analysis data (gauge, radar and satellite). **a**, **c** are CC, **b**, **d** are root-mean-square (RMS) (mm/h). Panels **a**, **b** are calculated on grid boxes with gauges, **c**, **d** are calculated on grid boxes without gauges

To verify the effectiveness of the compound merging strategy in the data merging, one year’s experimental UKGrsHP over 2014 is now produced. The differences between the experimental UKGrsHP and the three input analysis datasets are quantified separately using the metrics of CC and RMSE (Fig. 8). At grid boxes with at least one gauge observation, the CC (Fig. 8a) is nearly 1 (0.99889) and the RMS (Fig. 8b) is nearly zero (0.01542) between the experimental UKGrsHP and the gauge analysis, demonstrating that the merged data at these grid boxes mainly represents the information from the gauge observations. In contrast, at grid boxes with no gauge observations, the CC (Fig. 8c) is greatest (0.95357) and the RMS (Fig. 8d) is smallest (0.12182) between the experimental UKGrsHP and the radar analysis among the three input analyses, demonstrating that the merged data at these grid boxes mainly represents information from the radar analysis data. In either case, the merged data contains the least information from the satellite analysis data among the three input analyses, as proved by the minimum CC (Fig. 8a, c) (0.19241 and 0.20421) and maximum RMS (Fig. 8b, d) (0.4377 and 0.50343) between the experimental UKGrsHP and the satellite analysis data. In addition, at grid boxes with neither gauge observations nor radar analysis data, only information from the satellite analysis data is used (figure not shown). So the information usage from the input data in the experimental UKGrsHP are generally according to the accuracy and availability of the input datasets, which is reasonable.

Figure 3e shows the spatial distribution of the experimental UKGrsHP at 12 Z on 1st February 2014 using the compound merging strategy. The final merged precipitation, i.e. the UKGrsHP, not only captures the high rainfall amount centers reported by the gauge analysis well, but also depicts a more detailed rain distribution like radar analysis data and has full spatial coverage like satellite analysis data. Comparing Fig. 3b and e, we can see that intense rainfall signals over Great Britain around 55°N in the gauge analysis data is captured well by the UKGrsHP. Comparing Fig. 3c and e, we can see the detailed spatial rainfall details in the Nimrod radar data are represented well in the UKGrsHP. Comparing Fig. 3d and e, we find the information for rainfall over ocean areas are also transmitted into the UKGrsHP. Basically, the final merged precipitation product extracts the useful information from the input analysis data, providing a better representation of the precipitation. Similar results can be verified for other randomly chosen precipitation events as shown in Sect. 1 of the Supplementary Information.

Comparing Fig. 3e with Fig. 6a, we find the spatial distribution of the merged precipitation produced using the compound merging strategy (Fig. 3e) is similar to that of the merged results using the full 3_Merge framework (Fig. 6a). This indicates that the compound merging strategy performs well and is in line with expectations from the full merging. Considering the compound merging strategy saves large computing resources, it is therefore reasonable to choose it in our OI–based multi-source merging scheme. Equally, the compound merging strategy is highly flexible, which is suitable for applying to other (or wider) regions where the availability of input data resources are limited.

From the above, we choose the compound merging strategy in our OI–based multi-source merging scheme for producing the UKGrsHP.

## Independent evaluation of the experimental UKGrsHP

To objectively assess the performance of the experimental UKGrsHP produced by the OI–based multi-source merging scheme (with the compound merging strategy) in this study, an independent evaluation is then conducted. One year’s experimental UKGrsHP over 2014 is produced. Different from the experimental UKGrsHP in the previous section that merges all gauge observations, here we produce a merged product without using the ~ 220 MIDAS gauge observations and then independently evaluate this product against the MIDAS observations. For hourly precipitation, previous studies have shown that 1 year’s sample size is big enough for effective evaluations (e.g., Shen et al. 2018; Pan 2012). We quantify the differences between the experimental UKGrsHP, the input precipitation analysis data (the gauge analysis, radar analysis and satellite analysis) and the ~ 220 MIDAS gauge observations using two metrics, the CC and the RMSE. The independent evaluation method is further described in Sect. 2.3.

### General errors

Table 1 shows the annual and monthly mean values of the CC and RMSE in the UKGrsHP and the three input precipitation analyses. From Table 1, we can see that the UKGrsHP has a smaller annual mean RMSE (0.265 mm/h) and higher annual mean CC (0.702) than any one of the three input analysis datasets (radar, satellite, gauge). This suggests that the experimental UKGrsHP also has a better accuracy than all individual input analyses. These results demonstrate the effectiveness of the OI–based multi-source merging scheme in constructing a merged precipitation analysis dataset for the UK.

**Table 1:** Monthly and annual mean CC and RMSE (mm/h) of three input precipitation analysis datasets and the experimental UKGrsHP against ~ 220 independent MIDAS gauge observations from January to December of 2014 over the UK

	Jan.	Feb.	Mar.	Apr.	May.	Jun.	Jul.	Aug.	Sep.	Oct.	Nov.	Dec.	Annual
*Correlation coefficient (CC)*													
Gauge	0.703	0.695	0.584	0.601	0.572	0.441	0.489	0.556	0.356	0.665	0.652	0.606	0.576
Radar	0.743	0.728	0.597	0.713	0.705	0.607	0.648	0.668	0.427	0.749	0.744	0.634	0.663
GSMaP	0.299	0.318	0.209	0.261	0.301	0.205	0.23	0.276	0.114	0.338	0.295	0.151	0.250
UKGrsHP	0.787	0.767	0.64	0.743	0.731	0.645	0.673	0.73	0.451	0.787	0.783	0.685	0.702
*Root-mean-square error (RMSE) (mm/h)*													
Gauge	0.388	0.375	0.225	0.23	0.323	0.276	0.337	0.459	0.14	0.419	0.338	0.276	0.315
Radar	0.371	0.378	0.227	0.208	0.273	0.223	0.279	0.445	0.127	0.395	0.304	0.282	0.293
GSMaP	0.724	0.64	0.345	0.341	0.48	0.348	0.435	0.693	0.176	0.625	0.503	0.445	0.480
UKGrsHP	0.337	0.35	0.214	0.189	0.253	0.211	0.26	0.364	0.119	0.359	0.272	0.256	0.265

Another common feature shown in Table 1 is that the errors in the radar analysis and the gauge analysis data are close to each other and are much smaller than the errors in the satellite analysis data. This is demonstrated by both the RMSE and the CC. This result demonstrates the rationale of choosing the radar analysis data as the first-guess field or even as the joint observation field but excluding the satellite analysis where possible in the merging scheme.

### Spatial distribution of errors and improvements in the UKGrsHP

**Fig. 9:**
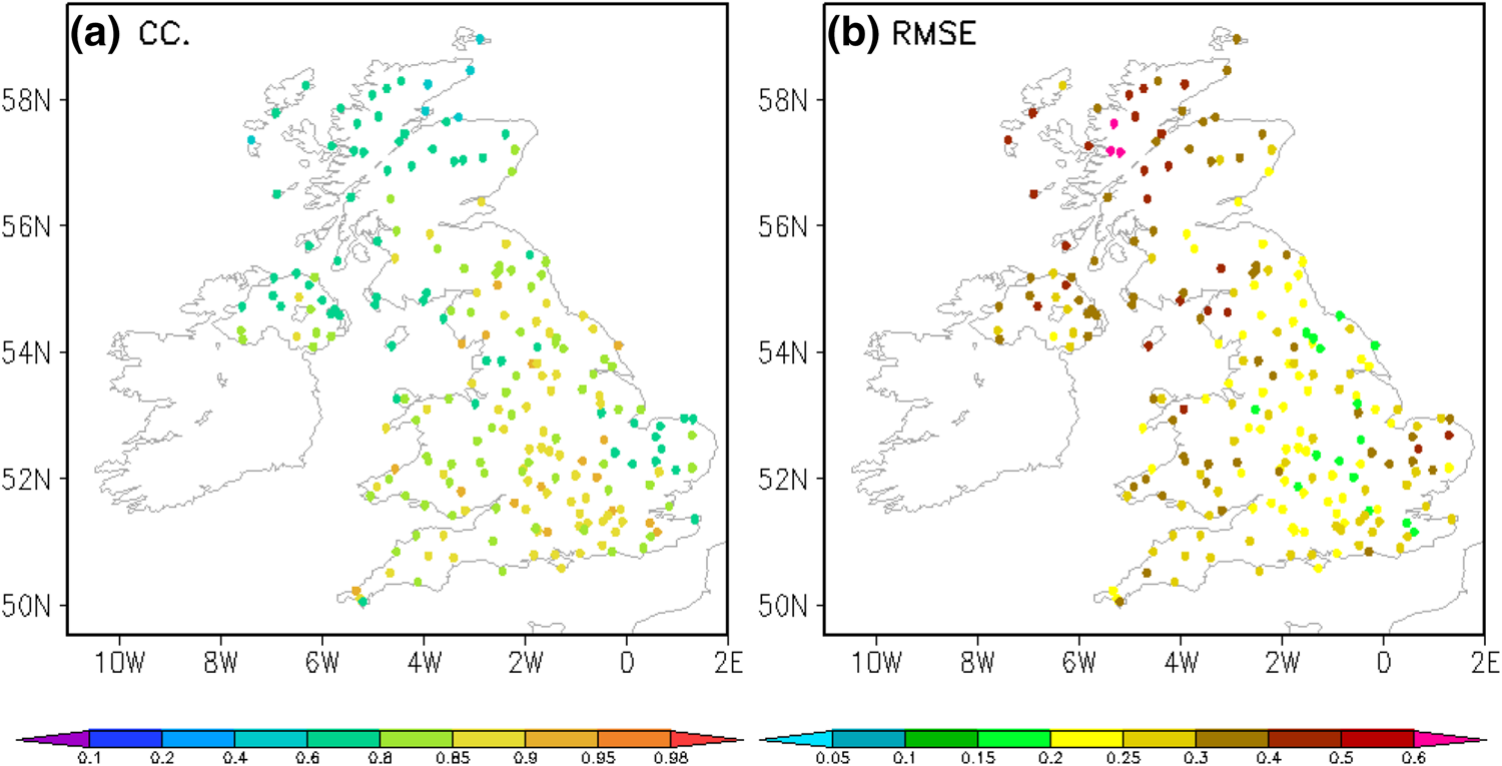
Spatial distribution of data errors for the merged results, the experimental UKGrsHP, against ~ 220 independent MIDAS gauge observations from January to December 2014 over the UK. Panels **a**, **b** are spatial distributions of CC and RMSE (mm/h), respectively

The spatial distribution of data errors in the experimental UKGrsHP are analyzed. Shown as Fig. 9, the CC (Fig. 9a) is generally higher and the RMSE (Fig. 9b) is lower in the southern UK than that in the northern UK, suggesting that the merged data has a better accuracy in the southern UK relative to that in the northern UK. Given that the density of the gauge observations in the southern UK is much greater than that in the northern UK (Fig. 1c), it suggests that the denser the gauge observations, the smaller the errors in the experimental UKGrsHP.

**Fig. 10:**
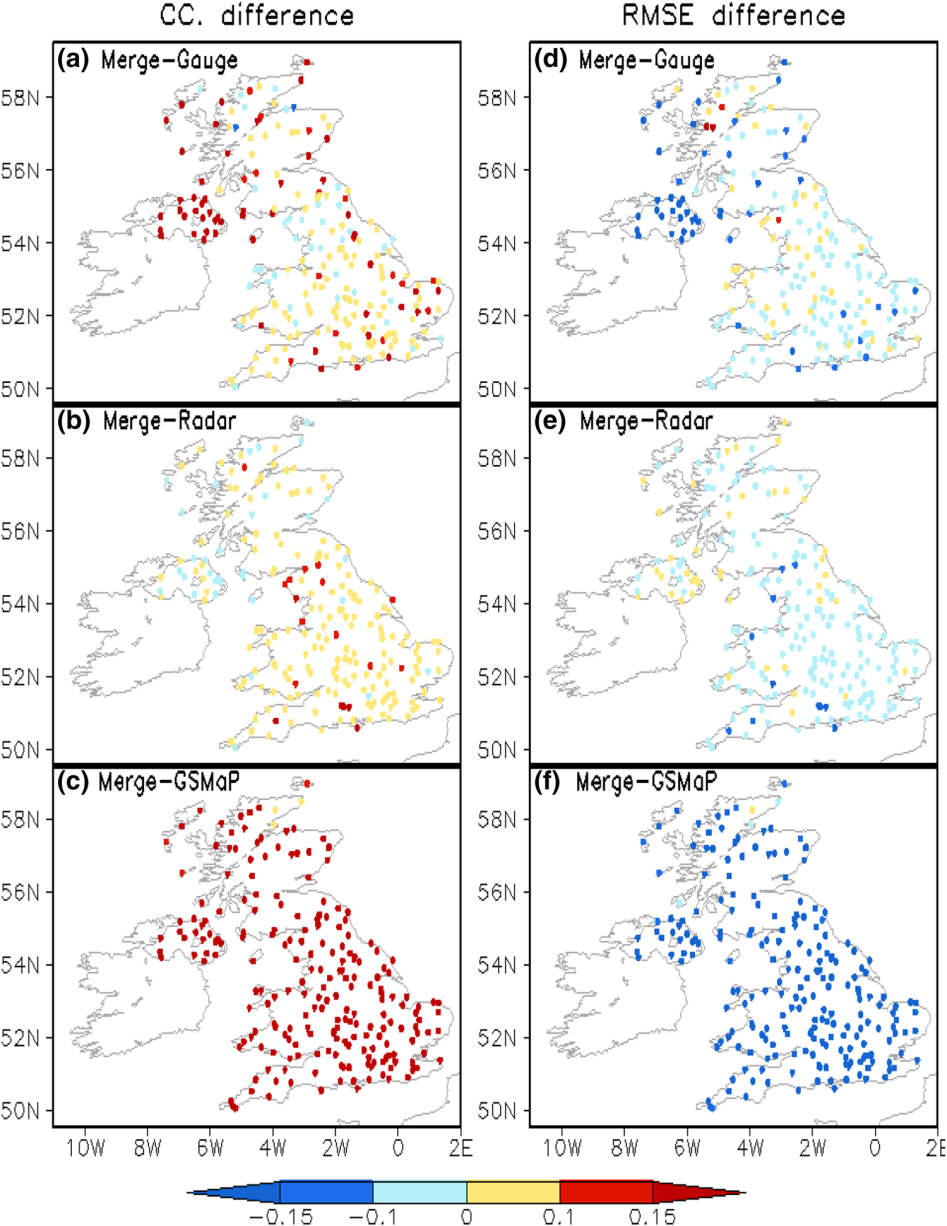
Spatial distribution of the difference between the data errors of the experimental UKGrsHP and the three input precipitation analyses. Left panels (**a**–**c**) are CC; right panels (**d**–**f**) are RMSE (mm/h). Panels **a**, **f** Are for gauge analysis, **b**, **e** are for radar analysis, **c**, **f** are for satellite analysis. The data error differences are calculated as CC and RMSE in the UKGrsHP minus that of the input analysis. Same as Fig. 9, the data errors in each analysis data are calculated against ~ 220 independent MIDAS gauge observations from January to December 2014 over the UK

To examine where the UKGrsHP is most improved relative to the individual input analysis data, we separately compare the data errors in the experimental UKGrsHP with that of the three input analyses. Figure 10 shows the spatial distribution of the difference between the data errors of the experimental UKGrsHP and the three input precipitation analyses. To begin with, we find that, over most parts of UK, the UKGrsHP is generally improved against the individual input analysis data. This is also proven by the UKGrsHP’s CC (RMSE) being higher (smaller) than that of the gauge analysis, radar analysis and satellite analysis in most parts of UK, as shown in Fig. 10a–c (Fig. 10d–f). We can see from Fig. 10 that the data error differences in the gauge and radar analysis data are relatively smaller than those of the satellite analysis data. This suggests that the improvement in accuracy of the UKGrsHP relative to the satellite analysis data is the largest among all the input analysis.

Relative to the gauge analysis data, the UKGrsHP exhibits obvious improvements over large areas where there is a low density of gauge observations. Over coastal areas of Britain with sparse gauge observations, the CC difference (Fig. 10d) is bigger than 0.1 and the RSME difference (Fig. 10d) is smaller than − 0.1. Since there are no gauges involved in the merging over Ireland, the merging result here is mainly controlled by the radar data, and is obviously superior to the gauge interpolation data. In other areas which have denser gauge observations, the improvements in UKGrsHP are not as obvious. Further analysis indicates that the improvements of the UKGrsHP in areas with sparse gauge observations are mainly due to the merging of the radar data.

Relative to the radar analysis data, the UKGrsHP exhibit obvious improvements over northeast England centered at 3°W, 54°N where there are very dense gauge observations. This is demonstrated by the difference in CC (Fig. 10b: > 0.1) and the RMSE (Fig. 10e: < − 0.1).

### Dependency of errors on precipitation intensity

The dependency of the errors on the precipitation intensity is also investigated. To assess the performance of the merging analysis in detecting and quantifying precipitation events of various intensities, we grouped the independent test data into six categories based on the hourly independent gauge precipitation intensity, then computed the Bias and RSME for each of the groups (Table 2). The number of samples are also shown for each group in Table 2, which are large enough to get representative results.

**Table 2:** The bias (mm/h) and RMSE (mm/h) for satellite, radar, gauge analysis data and experimental UKGrsHP data based on different precipitation intensity bins (including statistics sampling numbers) against independent gauge stations for January to December of 2014 over the UK

Pre. Intensity	Bias (mm/h)				RMSE (mm/h)				Sampling numbers
	GSMaP	Radar	Gauge	UKGrsHP	GSMaP	Radar	Gauge	UKGrsHP	
0	0.01899	0.02454	0.02844	0.02092	0.19189	0.15031	0.1645	0.1086	1,573,976
(0,1]	− 0.1874	0.02889	− 0.03861	0.02372	0.80616	0.47581	0.46212	0.4523	201,597
(1,2]	− 0.78926	− 0.25135	− 0.42845	− 0.19204	1.75492	0.92129	0.94838	0.86907	37,972
(2,4]	− 1.59026	− 0.74875	− 1.00597	− 0.58688	2.96402	1.48888	1.64283	1.36843	19,409
(4,8]	− 3.46584	− 2.00986	− 2.31417	− 1.60644	5.28602	2.84067	3.1934	2.54952	4697
(8, + ∞)	− 8.63316	− 6.13796	− 6.62829	− 5.11348	12.72729	7.30828	8.33925	6.42511	508

A remarkable feature in Table 2 is that the all three input analysis datasets and the UKGrsHP overestimate the intensity of light rainfall, but underestimate the heavy precipitation. This is evidenced by their positive Bias in light precipitation rates below 1 mm h^−1^, and their negative Bias for rainfall heavier than 1 mm h^−1^. Data errors in the merged data and three input datasets generally increase with precipitation intensity, with increasing RMSE from light rainfall to heavy rainfall for all three input analyses and for the UKGrsHP shown in Table 2. These are two common features in various precipitation data (e.g., Xie and Xiong 2011; Yu et al. 2013; Shen et al. 2014; Li et al. 2015).

However, we emphasize that the UKGrsHP contains the least Bias and the lowest RMSE in all precipitation groups above 0 mm h^−1^. Therefore, the data merging process has not only suppressed the systematic bias but also reduced the random errors, and the final merged product, the UKGrsHP, has a better accuracy than any one of the three input precipitation analyses for the precipitation intensity bins.

## Conclusions and discussion

An OI–based multi-source merging scheme with compound strategy has been developed to produce a merged high-resolution precipitation analysis dataset over the UK—the UKGrsHP. One year (from 1 January to 31 December 2014) of experimental UKGrsHP is produced in this study, which is at one hour time step with a temporal resolution of 0.01° × 0.01°, covering the wider UK region (12.5° W–3.5° E, 49° N–60° N). As depicted in the flowchart of the OI–based multi-source merging scheme in Fig. 2, we have three major merging steps: data preprocessing, bias removal and combining multi-source data. First, various data are all processed to the same spatial–temporal resolution (0.01º × 0.01º, hourly) by interpolating or scaling transformation. Then, bias correction is performed for the radar/satellite precipitation analysis values by matching the PDF (Yu et al. 2013) of the radar/satellite analysis data with that of the gauge analysis. After the PDF systematic bias-removal process, biases in the radar analysis and satellite GSMaP analysis data were significantly reduced. Then, the OI-based multi-source merging scheme with compound strategy was applied to combine the bias-corrected radar and satellite analysis data with the gauge analysis data. Finally, an independent test for 1 year (1 January to 31 December 2014) of the experimental UKGrsHP was conducted to verify the merging procedures and final merged results.

The employment of a compound strategy in our data merging scheme is mainly attributed to the large errors in the satellite analysis data, which causes non-substantial improvements in the quality of the UKGrsHP after merging the satellite analysis. However, over oceanic areas without coverage of either the gauge or the radar analysis data, the satellite analysis data is still valuable. Considering the gauge-satellite-radar (or the 3_Merge) framework uses much more computing resources than the compound strategy, it is also economical to choose the compound strategy. In addition, the compound strategy of using the gauge-satellite-radar (3_Merge) framework and the gauge-radar (2_Merge) framework provides flexibility in application to precipitation merging in other regions.

We have demonstrated that the OI-based multi-source merging scheme developed in this study is effective and can enhance the advantages of individual precipitation measurements. The final merged precipitation analysis—the UKGrsHP—has complete spatial coverage like satellite data, retains high spatial resolution like radar data, and shows a better accuracy than any single-source input precipitation analysis product. A full version of the UKGrsHP starting from April 2004 is now under development.

There are two ways to improve the quality of merged precipitation products, including the UKGrsHP, in the future. One is to improve the quality of individual rainfall sources, which is fundamental. The gauge data is vital for improving the accuracy of the merged data, so we need to collect more and denser gauge observations and apply better quality-controls on these gauges. Currently, we’re trying to collect Irish gauges, which we hope will be added to our merging data in the future. Then, the innovation and replacement of observation instruments inevitably will bring about the improvement of precipitation observation data. For example, the Global Precipitation Measurement (GPM) mission is an international network of satellites launched in 2014 that provides next-generation global observations of rain and snow, which build upon the success of the Tropical Rainfall Measuring Mission (TRMM). GPM is supposed to capture light rainfall and snowfall more accurately than TRMM and other existing satellite precipitation products. As light rainfall and snowfall are very important precipitation types in middle and high latitudes, GPM products may be an important source of data for improving the quality of high-latitude merged datasets in the future, including our UKGrsHP. Moreover, with the development of techniques in numerical simulation and data assimilation, we can take advantage of information from numerical weather or hydrological models in the future, especially in regions with sparse observations or during cold seasons (Ebert et al. 2007).

The other way is to develop better merging algorithms. The future development of objective algorithms for precipitation analysis needs to incorporate information from all available sources, including gauge measurements, radar observations, satellite estimates and even model simulations/forecasts, which also need to examine the impacts of including/withdrawing input observations in order to achieve the best balance between homogeneity in the long-term time series and the quantitative.

## Electronic supplementary material

Below is the link to the electronic supplementary material.

Supplementary material 1 (PDF 279 kb)

## References

[CR1] Adler RF, Kidd C, Petty G, Morrisey M, Goodman MH (2001). Intercomparison of global precipitation products: the Third Precipitation Intercomparison Project (PIP-3). Bull Am Meteorol Soc.

[CR2] Adler RF (2003). The version-2 global precipitation climatology project (GPCP) monthly precipitation analysis (1979–present). J Hydrometeorol.

[CR3] Archer DR, Fowler HJ (2015). Characterising flash flood response to intense rainfall and impacts using historical information and gauged data in Britain. J. Flood Risk Manage.

[CR4] Barth A, Azcárate AA, Joassin P, Jean-Marie B, Troupin C (2008). Introduction to optimal interpolation and variational analysis. Presented at the SESAME summer school.

[CR5] Beck HE, van Dijk AIJM, Levizzani V, Schellekens J, Miralles DG, Martens B, de Roo A (2017). MSWEP: 3-hourly 0.25 degrees global gridded precipitation (1979–2015) by merging gauge, satellite, and reanalysis data. Hydrol Earth Syst Sci.

[CR6] Beck HE, Wood EF, Pan M, Fisher CK, Miralles DG, van Dijk AIJM, McVicar TR, Adler RF (2019). MSWEP V2 global 3-hourly 0.1° precipitation: methodology and quantitative assessment. Bull Am Meteorol Soc.

[CR7] Bell TL, Kundu PK (2000). Dependence of satellite sampling error on monthly averaged rain rates: comparisons of simple models and recent studies. J Clim.

[CR8] Bergman KH (1978). Role of observational errors in optimum interpolation analysis. Bull Am Meteorol Soc.

[CR9] Blenkinsop S, Lewis E, Chan SC, Fowler HJ (2017). Quality-control of an hourly precipitation dataset and climatology of extremes for the UK. Int J Climatol.

[CR10] Blenkinsop S, Fowler HJ, Barbero R, Chan SC, Guerreiro SB, Kendon E, Lenderink G, Lewis E, Li X-F, Westra S, Alexander L, Allan RP, Berg P, Dunn RJH, Ekström M, Evans J, Holland G, Jones R, Kjellström E, Klein-Tank A, Lettenmaier D, Mishra V, Prein AF, Sheffield J, Tye MR (2018). The INTENSE project: using observations and models to understand the past, present and future of sub-daily rainfall extremes. Adv Sci Res.

[CR11] Boushaki FI, Hsu KL, Sorooshian S, Park GH, Mahani A, Shi W (2009). Bias adjustment of satellite precipitation estimation using ground-based measurement: a case study evaluation over the southwestern United States. J Hydrometeorol.

[CR12] Brankart J-M, Brasseur P (1996). Optimal analysis of in situ data in the Western Mediterranean using statistics and cross-validation. J Atmos Oceanic Technol.

[CR13] Cai Y, Jin C, Wang A, Guan D, Wu J, Yuan F, Xu L (2015). Spatio-temporal analysis of the accuracy of tropical multisatellite precipitation analysis 3B42 precipitation data in mid-high latitudes of China. PLoS One.

[CR14] Chen M, Shi W, Xie P, Silver VB, Kousky VE, Janowiak JE (2008). Assessing objective techniques for gauge-based analyses of global daily precipitation. J Geophys Res.

[CR15] Chiang Y-M, Hsu K-L, Chang F-J, Hong Y, Sorooshian S (2007). Merging multiple precipitation sources for flash flood forecasting. J Hydrol.

[CR16] Chumchean S, Seed A, Sharma A (2006). Correcting of real-time radar rainfall bias using a kalman filtering approach. J Hydrol.

[CR17] Dai A, Lin X, Hsu KL (2007). The frequency, intensity, and diurnal cycle of precipitation in surface and satellite observations over lowand mid-latitudes. Clim Dyn.

[CR18] Daly R (1991). Atmospheric data analysis.

[CR19] Ebert EE (1996) Results of the 3rd Algorithm Intercomparison Project (AIP-3) of the Global Precipitation Climatology Project (GPCP), Revision 1. Bureau of Meteorology Research Centre, p 199

[CR20] Ebert EE, Janowiak JE, Kidd C (2007). Comparison of near-realtime precipitation estimates from satellite observations and numerical models. Bull Am Meteorol Soc.

[CR21] Gandin LS (1965) Objective analysis of meteorological fields. Israel Program for Scientific Translation, Jerusalem, p 242

[CR22] Gibson M (2000). Comparative study of several gauge adjustment schemes. Phys Chem Earth Part B.

[CR23] Golding B (1998). Nimrod: a system for generating automated very short range weather forecasts. Meteorol Appl.

[CR24] Habib E, Ciach GJ (2001). Estimation of rainfall interstation correlation. J Hydrometeorol.

[CR25] Haiden T, Pistotnik G (2009). Intensity-dependent parameterization of elevation effects in precipitation analysis. Adv Geosci.

[CR26] Haiden T, Kann A, Wittmann C, Pistotnik G, Bica B, Gruber C (2011). The integrated nowcasting through comprehensive analysis (INCA) system and its validation over the Eastern Alpine region. Weather Forecast.

[CR27] Harrison D, Driscoll S, Kitchen M (2000). Improving precipitation estimates from weather radar using quality control and correction techniques. Meteorol Appl.

[CR28] Hong Y, Gochis D, Cheng J-T, Hsu KL, Sorooshian S (2007). Evaluation of PERSIANN-CCS rainfall measurement using the NAMEevent rain gauge network. J Hydrometeorol.

[CR29] Huffman GJ (1997). Estimates of root-mean-square random error for finite samples of estimated precipitation. J Atmos Sci.

[CR30] Huffman GJ, Adler RF, Morrissey MM, Bolvin DT, Curtis S, Joyce R, McGavock B, Susskind J (2001). Global precipitation at one-degree daily resolution from multisatellite observations. J Hydrometeorol.

[CR31] Kalnay E (2002). Atmospheric modeling, data assimilation, and predictability.

[CR32] Kendon EJ, Roberts NM, Fowler HJ, Roberts MJ, Chan SC, Senior CA (2014). Heavier summer downpours with climate change revealed by weather forecast resolution model. Nat Clim Change.

[CR33] Lewis E, Quinn N, Blenkinsop S, Fowler HJ, Freer J, Tanguy M, Hitt O, Coxon G, Bates P, Woods R (2018). A rule based quality control method for hourly rainfall data and a 1 km resolution gridded hourly rainfall dataset for Great Britain: cEH-GEAR1hr. J Hydrol.

[CR34] Lewis E, Fowler HJ, Alexander L, Dunn R, McClean F, Barbero R, Guerreiro S, Li X-F, Blenkinsop S (2019). GSDR: a global sub-daily rainfall dataset. J Clim.

[CR35] Li Q, Ferraro R, Grody N (1998). Detailed analysis of the error associated with the rainfall retrieved by the NOAA/NESDIS SSM/I algorithm 1: tropical oceanic rainfall. J Geophys Res.

[CR73] Li H, Hong Y, Xie P, Gao J, Niu Z, Kirstetter P, Yong B (2015). Variational merged of hourly gauge-satellite precipitation in China: preliminary results. J Geophys Res Atmos.

[CR36] Lin Y, Mitchell KE (2005) The NCEP stage II/IV hourly precipitation analyses: development and applications, 19th Conference on Hydrology, American Meteorological Society, San Diego

[CR37] Maddox RA, Zhang J, Gourley JJ, Howard KW (2002). Weather radar coverage over the contiguous United States. Weather Forecast.

[CR38] Makihara Y (2000). Algorithms for precipitation nowcasting focused on detailed analysis using radar and raingauge data, Study on the Objective Forecasting Techniques. Tech Rep Meteorol Res Inst.

[CR39] McMillan H, Krueger T, Freer J (2012). Benchmarking observational uncertainties for hydrology: rainfall, river discharge and water quality. Hydrol Process.

[CR40] Morrissey ML, Maliekal JA, Greene JS, Wang J (1995). The uncertainty of simple averages using rain gauge networks. Water Resour Res.

[CR41] Ordnance Survey (2008) A Guide to Coordinate Systems in Great Britain: an introduction to mapping coordinate systems and the use of GPS datasets with Ordnance Survey mapping.Ordnance Survey, Southampton, UK

[CR42] Pan Y, Shen Y, Yu J, Zhao P (2012). Analysis of the combined gauge-satellite hourly precipitation over China based on the OI technique. Acta Meteorologica Sinica.

[CR43] Pan Y, Shen Y, Yu J, Xiong A (2015). An experiment of high-resolution gauge–radar–satellite combined precipitation retrieval based on the Bayesian merging method. Acta Meteorologica Sinica.

[CR44] Pan Y, Gu J, Yu J, Shen Y, Shi C, Zhou Z (2018). Test of merging methods for multi-source observed precipitation products at high resolution over China. Acta Meteorologica Sinica.

[CR45] Parkes B, Wetterhall F, Pappenberger F, He Y, Malamud B, Cloke H (2013). Assessment of a 1-hour gridded precipitation dataset to drive a hydrological model: a case study of the summer 2007 floods in the Upper Severn, UK. Hydrol Res.

[CR46] Seo DJ, Breidenbach JP (2002). Real-time correction of spatially nonuniform bias in radar rainfall data using rain gauge measurements. J Hydrometeorol.

[CR47] Serreze MC, Andrew PB, Lo F (2005). Northern high-latitude precipitation as depicted by atmospheric reanalyses and satellite retrievals. Mon Weather Rev.

[CR48] Shen Y, Xiong A, Wang Y, Xie P (2010). Performance of high-resolution satellite precipitation products over China. J Geophys Res.

[CR49] Shen Y, Feng M, Zhang H, Gao X (2010). Interpolation methods of China daily precipitation data. J Appl Meteorol Sci.

[CR50] Shen Y, Zhao P, Pan Y, Yu J (2014). A high spatiotemporal gauge-satellite merged precipitation analysis over China. J Geophys Res Atmos.

[CR51] Shen Y, Hong Z, Pan Y, Yu J, Maguire L (2018). China’s 1 km merged gauge, radar and satellite experimental precipitation dataset. Remote Sens.

[CR52] Simanton JR, Osborn HB (1980). Reciprocal-distance estimate of point rainfall. J Hydraul Div.

[CR53] Smith JA, Krajewski WF (1991). Estimation of the mean field bias of radar rainfall estimates. J Appl Meteorol.

[CR54] Storch HV, Zwiers FW (1999). Statistical analysis in climate research.

[CR55] Sun Q, Miao C, Duan Q, Ashouri H, Sorooshian S, Hsu KL (2018). A review of global precipitation data sets: Data sources, estimation, and intercomparisons. Rev Geophys.

[CR56] Surface Stations Data (1853) NCAS British Atmospheric Data Centre, last accessed 14 June 2015. http://catalogue.ceda.ac.uk/uuid/220a65615218d5c9cc9e4785a3234bd0

[CR57] Taylor J (1997). Introduction to error analysis, the study of uncertainties in physical measurements.

[CR58] Tian Y, Peters-Lidard CD, Eylander JB, Joyce RJ, Huffman GJ, Adler RF, Hus K-L, Turk FJ, Garcia M, Zeng J (2009). Component analysis of errors in satellite-based precipitation estimates. J Geophys Res.

[CR59] UK Met Office, 2012: Met Office Integrated Data Archive System (MIDAS) Land and Marine

[CR60] UK Meteorological Office. Rain radar Products (NIMROD), [Internet]. British Atmospheric Data Centre, 2003: Date of citation. http://badc.nerc.ac.uk/data/nimrod

[CR61] Ushio T (2009). A Kalman filter approach to the Global Satellite Mapping of Precipitation (GSMaP) from combined passive microwave and Infrared radiometric data. J Meteorol Soc Jpn.

[CR62] Vasiloff SV (2007). Improving QPE and very short term QPF. Bull Am Meteorol Soc.

[CR63] Westra S, Fowler HJ, Evans JP, Alexander LV, Berg P, Johnson F, Kendon EJ, Lenderink G, Roberts NM (2014). Future changes to the intensity and frequency of short-duration extreme rainfall. Rev Geophys.

[CR64] Xie P, Arkin P (1997). Global precipitation: a 17-year monthly analyses based on gauge observations, satellite estimates, and numerical model outputs. Bull Am Meteorol Soc.

[CR65] Xie P, Xiong A (2011). A conceptual model for constructing high-resolution gauge-satellite merged precipitation analyses. J Geophys Res Atmos.

[CR66] Xie P, Yatagai A, Chen M, Hayasaka T, Fukushima Y, Liu C, Yang S (2007). A gauge-based analysis of daily precipitation over East Asia. J Hydrometeorol.

[CR72] Xu B, Xie P (2010) Quantifying error in the CMORPH satellite precipitation estimates. Abstract H14A‐06 presented at 2010 Fall Meeting. AGU, San Francisco, California, 13–17 December

[CR67] Yin X, Gruber A, Arkin P (2004). Comparison of the GPCP and CMAP merged gauge-satellite monthly precipitation products for the period 1979–2001. J Hydrometeorol.

[CR68] Yu J, Shen Y, Pan Y, Zhao P, Zhou Z (2013). Improvement of satellite-based precipitation estimates over China based on probability density function matching method. J Appl Meteorol Sci.

[CR69] Yu J, Shen Y, Pan Y, Xiong A (2015). Comparative assessment between the daily merged precipitation dataset over China and the world’s popular counterparts. Acta Meteorologica Sinica.

[CR70] Zhang J (2011). National mosaic and multi-sensor QPE (NMQ) System description, results, and future plans. Bull Am Meteorol Soc.

[CR71] Zhang J, Howard K, Langston C, Kaney B, Qi Y, Tang L (2016). Multi-radar multi-sensor (MRMS) quantitative precipitation estimation: initial operating capabilities. Bull Am Meteorol Soc.

