# Supplementary material for: UKGrsHP: a UK high-resolution gauge–radar–satellite merged hourly precipitation analysis dataset
Source: Clim Dyn. 2020 Feb 7;54(5):2919–40. doi: 10.1007/s00382-020-05144-2 (PMC7089634; doi:10.1007/s00382-020-05144-2)
Supplement: Supplementary file 1 — Supplementary material 1 (PDF 279 kb) [file 382_2020_5144_MOESM1_ESM.pdf]

UKGrsHP: A UK High-Resolution Gauge-Radar-Satellite Merged Hourly Precipitation Analysis Dataset

Jingjing Yu[*], Xiao-Feng Li, Elizabeth Lewis, Stephen Blenkinsop, Hayley J. Fowler

School of Engineering, Newcastle University, Newcastle upon Tyne NE1 7RU, UK

[*] ***Corresponding author address***: Dr. Jingjing Yu, School of Engineering, Newcastle University, Newcastle upon Tyne NE1 7RU, UK
E-mail: Jingjing.Yu@newcastle.ac.uk

Supplementary Information

1. Examples of final UKGrsHP

We show here are 4 examples (Figure S1-S4) of the merged hourly data for precipitation events as additional examples to Figure 3. These examples are randomly chosen within wet events across the different seasons in 2014, including 09Z on 5th April (Figure S1), 03Z on 5th July (Figure S2), 23Z on 30th September (Figure S3), and 12Z on 22nd December (Figure S4), respectively.

Similarly to Figure 3, the four random examples (Figure S1-S4) provided here show that all 4 precipitation products (the gauge analysis, the radar analysis, the satellite analysis and the merged precipitation) can capture the basic features of the rainfall field in wet events. Gauge analysis data (Figure S1a, S2a, S3a, S4a) based on the interpolation of sparse gauges has limitations in giving a reliable and detailed spatial estimation of hourly rainfall, especially over areas without rain gauges. The radar analysis data (Figure S1b, S2b, S3b, S4b) provides better spatial pattern information, but has rainfall amount bias compared to the gauge analysis data, and still experiences incomplete spatial coverage. Moreover, we can also see some misrepresentations of land precipitation spatial patterns or large errors of satellite precipitation over land areas in the satellite analysis data (Figure S1c, S2c, S3c, S4c).

Generally, the UKGrsHP (Figure S1d, S2d, S3d, S4d) depicts a more spatially detailed rainfall distribution like the radar analysis data and full spatial coverage like the satellite analysis data. We also find that its rainfall amount values are close to the gauge analysis data. These random examples support the conclusion that the merged rainfall examples reflect the characteristics of the high-resolution precipitation product.

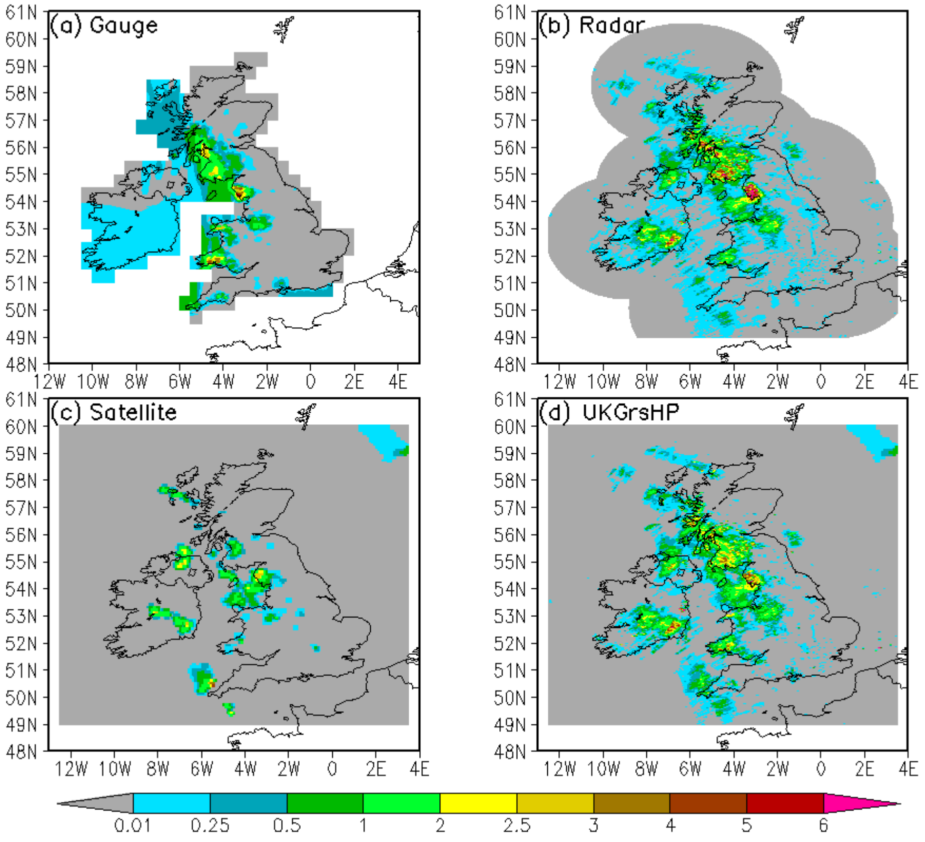

Figure S1 Spatial distribution of hourly precipitation (mm/h) at 09Z, 5th April 2014. (a) gauge analysis data interpolated from 1903 rain gauge observations, (b) NIMROD radar analysis data, (c) GSMaP satellite analysis data, and (d) the merged product, i.e. the experimental UKGrsHP. The experimental UKGrsHP is merged from the 1903 gauge observations, the NIMROD radar analysis and the GSMaP satellite analysis.

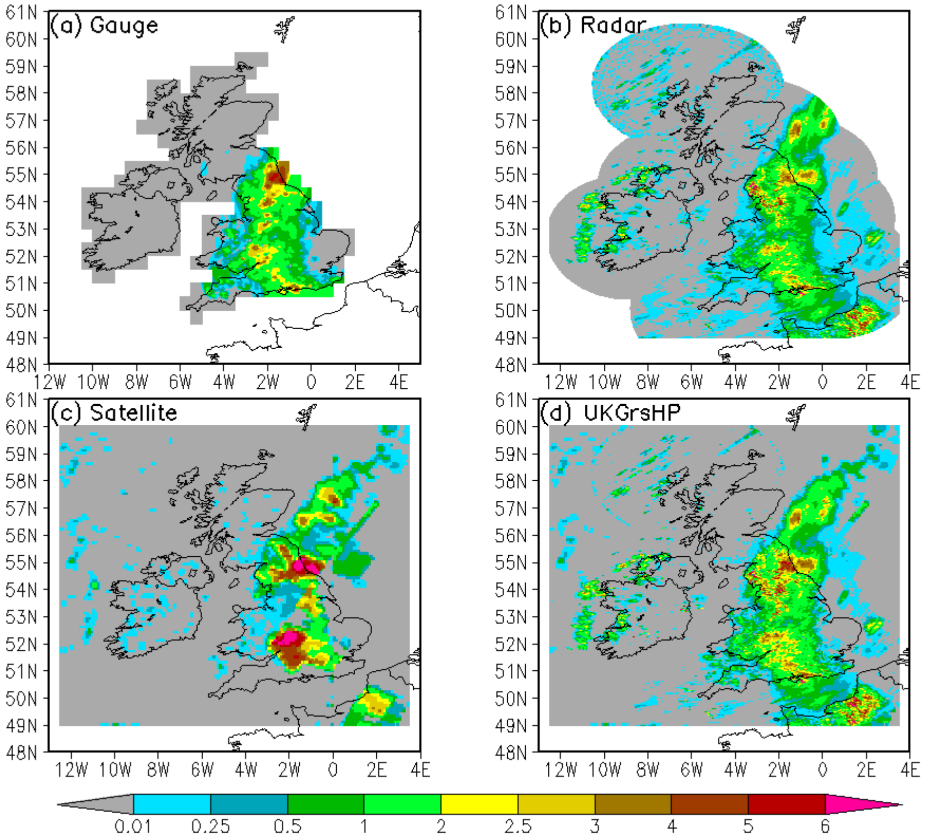

Figure S2 The same as Figure S1, but for 03Z, 5th July 2014.

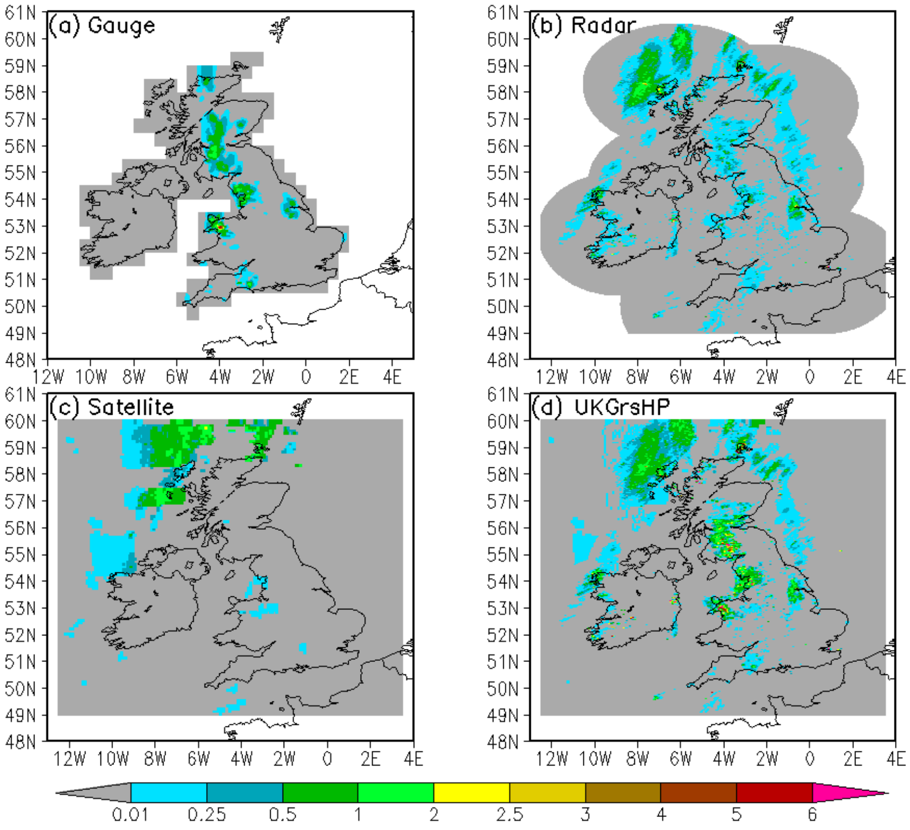

Figure S3 The same as Figure S1, but for 23Z, 30th September 2014.

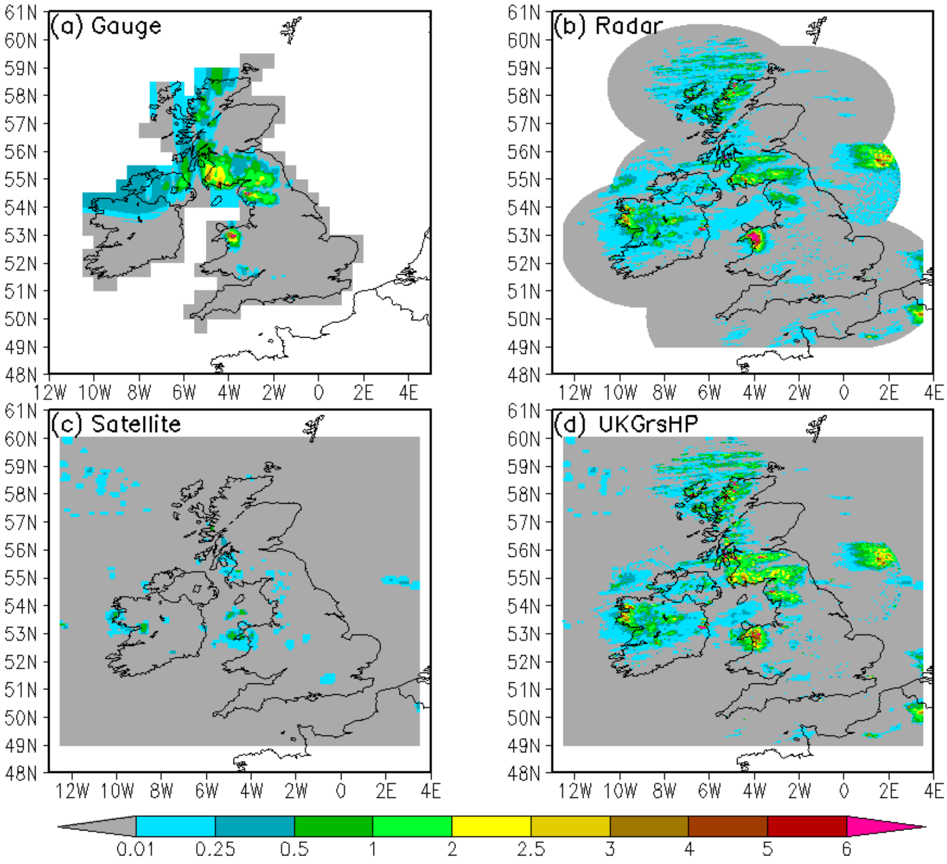

Figure S4 The same as Figure S1, but for 12Z, 22nd December 2014.

2. Examples of merged precipitation using two different merging schemes

These are 4 extracted examples (Figures S5-S8) of the merged hourly data in precipitation events additional to that shown in Figure 6, which are produced by the two merging frameworks. We use the same examples as for Figures S1-S5 in the previous section. All 4 examples demonstrate that the merged precipitation from the first scheme (3_Merge) and the second scheme (2_Merge) are generally similar to each other.

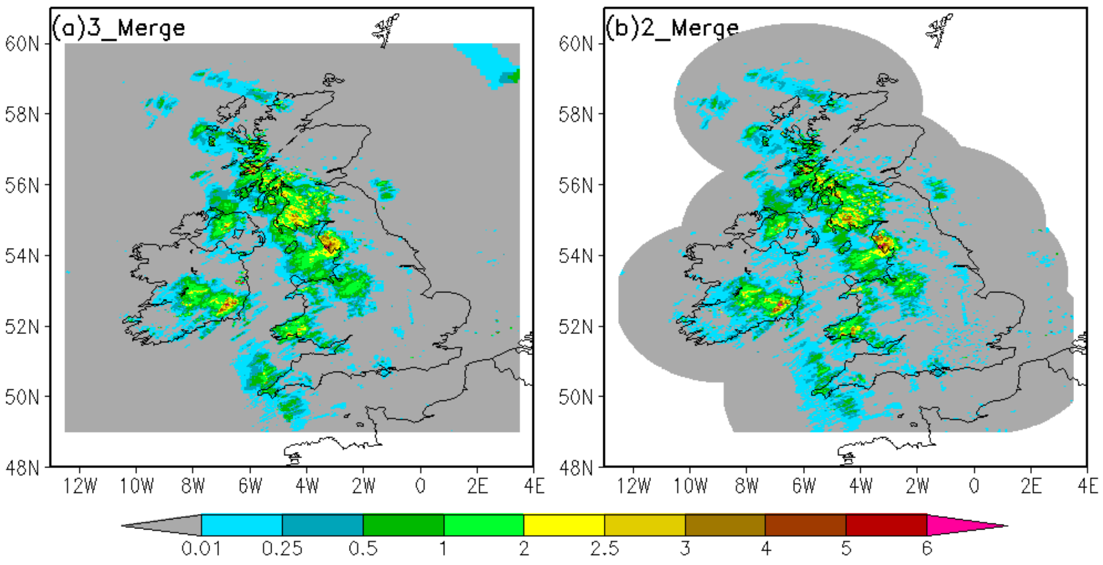

Figure S5 Spatial distribution of hourly merged precipitation (mm/h) using (a) 3_Merge and (b) 2_Merge framework at 09Z, 5th April 2014.

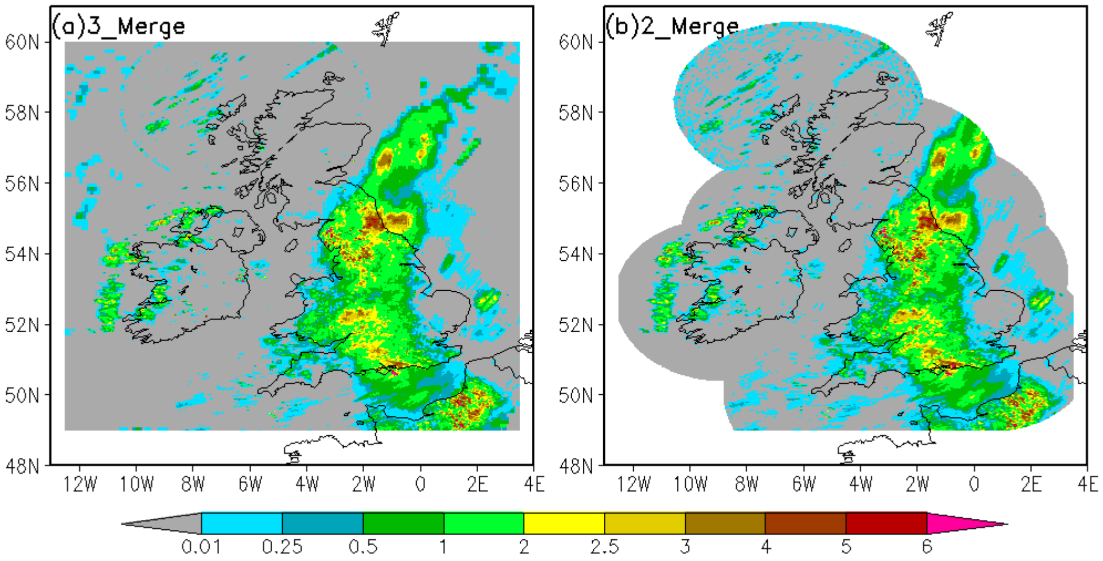

Figure S6 The same as Figure S5, but for 03Z, 5th July 2014.

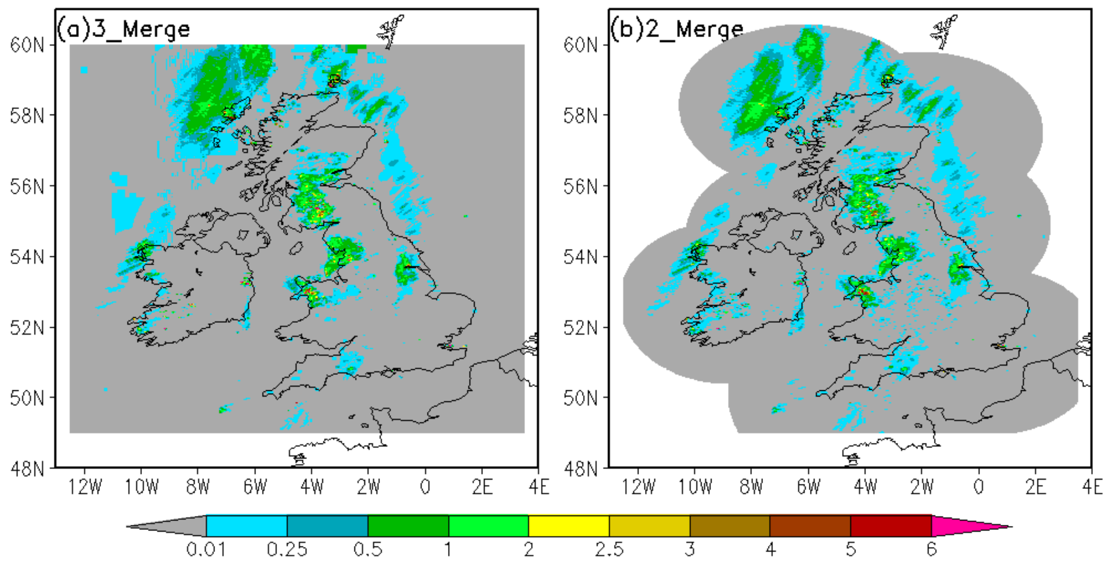

Figure S7 The same as Figure S5, but for 23Z, 30th September 2014.

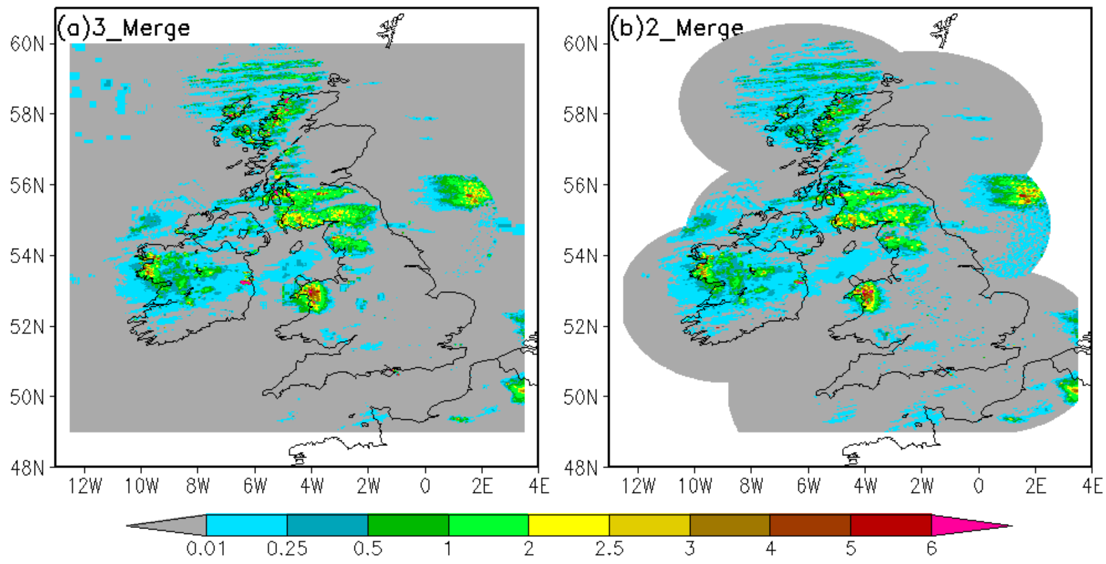

Figure S8 The same as Figure S5, but for 12Z, 22nd December 2014.
